# References values and standardized testing protocols for performance-based and patient-reported outcome measures among individuals with lower limb amputation

**DOI:** 10.3389/fresc.2026.1786298

**Published:** 2026-03-13

**Authors:** Matthew McGuire, Sara Nataletti, Rachel Maronati, Shenan Hoppe-Ludwig, Anushua Banerjee, Amber Wacek, Juan Cave, John M. Looft, Christopher L. Dearth, Brad D. Hendershot, Arun Jayaraman

**Affiliations:** 1 Max Näder Center for Rehabilitation Technologies and Outcomes Research, Shirley Ryan AbilityLab, Chicago, IL, United States; 2 Department of Physical Medicine & Rehabilitation, Northwestern University, Chicago, IL, United States; 3 Motion Analysis Laboratory, Department of Prosthetics, Minneapolis Veterans Affairs Health Care System, Minneapolis, MN, United States; 4 Rehabilitation & Engineering Center for Optimizing Veteran Engagement & Reintegration (RECOVER), Department of Research, Minneapolis Veterans Affairs Health Care System, Minneapolis, MN, United States; 5 Division of Rehabilitation Science, University of Minnesota Medical School, Minneapolis, MN, United States; 6 Extremity Trauma and Amputation Center of Excellence, Defense Health Agency, Falls Church, VA, United States; 7 Department of Rehabilitation, Walter Reed National Military Medical Center, Bethesda, MD, United States; 8 Department of Physical Medicine & Rehabilitation, Uniformed Services University of the Health Sciences, Bethesda, MD, United States; 9 Department of Physical Therapy & Human Movement Sciences, Northwestern University, Chicago, IL, United States

**Keywords:** clinical outcome measures, K-levels, lower-limb amputation, prosthesis, reference values, rehabilitation

## Abstract

**Introduction:**

Reliable reference data and standardized administration procedures for outcome measures are critical to guide rehabilitation, justify prosthetic prescription, and evaluate treatment outcomes in individuals with lower limb amputation (LLA). Although numerous performance-based and patient-reported measures are used in this population, few studies provide both population-specific reference values and detailed testing protocols. This study describes standardized administration procedure and presents reference values for commonly used outcome measures categorized by functional K-level and amputation level to support consistency in clinical and research applications.

**Materials and methods:**

Data was collected from the initial visit of a larger longitudinal study across three medical research centers. Participants included 58 adults (aged 18–89) with unilateral or bilateral transtibial or transfemoral amputation, all using at least one definitive prosthesis for ≥6 months and classified at K2 level or higher. Participants completed 8 performance-based and 11 patient-reported outcome measures.

**Results:**

Data was reported by K-level and amputation level (unilateral transtibial, unilateral transfemoral, bilateral). Some discrepancies were observed in various assessments compared to prior studies, possibly due to methodological and demographic differences. 10 Meter Walk Test speeds reported here were up to 31% faster than previous studies, potentially due to differences in track length and calculation methods.

**Conclusion:**

This study provides standardized administration guidelines and population-specific reference values for widely used performance-based and patient-reported outcomes in adults with LLA. Although these data are not intended to establish formal normative values due to limited sample size, they provide clinically relevant benchmarks for contextualizing individual performance, and support goal setting. By detailing test procedures and observed score distributions, these findings promote methodological transparency, improve comparability across studies, and support evidence-based prosthetic care and rehabilitation.

**Clinical Trial Registration:**

https://clinicaltrials.gov/study/NCT03930199, identifier NCT03930199.

## Introduction

Lower limb amputation (LLA) often adversely impacts mobility, demanding a multidisciplinary rehabilitation approach with reliable performance-based and patient-reported outcome benchmarks to guide clinical care and track improvements. Establishing normative values for LLA outcomes is crucial for advancing rehabilitative medicine and enhancing clinical care in prosthetics (1, 2) These benchmarks help healthcare professionals set realistic rehabilitation goals and expectations for improved functioning and quality of life. By identifying a standard range for individuals in similar conditions, clinicians can tailor rehabilitation programs to be both achievable and challenging.

Normative values are essential for clinicians to justify prosthetic prescriptions and advocate for reimbursement. They provide the empirical data to demonstrate the impact of prosthetic use and support the case for investment based on anticipated health outcomes. In clinical research, normative values offer a reference for assessing the efficacy of new treatments, prosthetic components, and therapeutic interventions/technologies, facilitating the advancement of evidence-based practices (3).

Despite their importance, LLA-specific normative values, typically derived from large, representative samples, are limited or unavailable for many commonly used outcome measures. Tools such as the Amputee Mobility Predictor Assessment Tool (AMP), the Comprehensive High Level Mobility Predictor (CHAMP), and the Prosthetic Limb User Survey for Mobility (PLUS-M) have been specifically designed and validated for the LLA population and have established normative values (4–7) However, widely used measures such as the Functional Gait Assessment (FGA) and the Patient-Reported Outcomes Measurement Information System (PROMIS) Mobility scale, were originally validated in older adult or neurologic populations, and although they are frequently used with prosthesis users, robust LLA-specific normative data are still lacking. This often leads to inappropriate comparison with the general population or other diagnoses, potentially adversely influencing prosthetic prescriptions, insurance reimbursements, and rehabilitation outcomes.

Finally, for normative values to be useful, both standardization and methodological transparency are vital. Standardized administration and scoring allow researchers and clinicians to report, share, and compare data across studies and clinical practices. Standardized assessments also enable data aggregation, meta-analyses, and systematic reviews enhancing conclusions about the effectiveness of therapeutic interventions and prosthetic components. However, when full standardization is not feasible or has not yet been universally adopted, clear and detailed reporting becomes essential. Transparent reporting helps contextualize the findings, ensures that observed differences across studies can be attributed to patient factors rather than methodological inconsistencies and allows others to assess the degree to which normative values are comparable across studies or clinical environments.

Efforts to address these gaps are ongoing, but significant data shortages remain, particularly regarding standardized procedures for administering and interpreting these measures. This study takes a first step toward addressing this need by providing population-specific reference values for commonly used performance-based and patient-reported outcome measures, together with clear documentation of the administration procedures followed at each site. Although subgroup sample sizes do not allow for the establishment of formal normative standards across all amputation and functional categories, the resulting reference values provide a practical starting point for interpreting clinical outcomes, supporting goal setting and facilitating comparison across research settings without implying normality. Ultimately, by reporting scores stratified by amputation and K level and by detailing how each measure was administered, this work supports more informed clinical decision-making and promotes greater methodological consistency in both research and clinical practice.

## Methods

### Participants

Sixty-five participants were recruited from 2020 to 2022 through research registries and prosthetics clinics of three medical research facilities within the scope of a larger clinical trial (ClinicalTrial.gov NCT03930199). The study was approved by the Institutional Review Board of each site, and all participants provided written informed consent.

Inclusion criteria were individuals aged 18–89 years with unilateral or bilateral LLA at the transtibial or transfemoral level, including knee disarticulations, who had been using at least one definitive prosthesis (i.e., a stable, non-preparatory prosthesis with a finished socket and componentry not anticipated to change) for at least six months. Participants were included if they had a designated Medicare Functional Classification Level, or equivalent, (K-Level) of K2-4 (i.e., at least a household ambulator). Exclusion criteria included co-morbidities that would limit prosthesis use, such as a cerebral vascular accident or severe traumatic brain injury, and unwillingness to use a study-related smartphone app.

### Data collection

In the broader study, participants engaged in up to 4 testing sessions, spaced three months apart, during each of which the same battery of 8 performance-based and 11 patient-reported assessments was administered. To avoid the effects of practice and exposure, the current report includes only data collected at the first session. To ensure measurement consistency within this study and in future comparisons, established protocols were followed for each measure as outlined in Appendix 1. Relevant pdf forms and scoring guides are provided in Supplementary Material 1.

### Performance-based outcome measures

The performance-based assessments were all completed in the research lab or clinic and included the Ten-Meter Walk Test (10MWT), Six-Minute Walk Test (6MWT), Five-Time Sit to Stand Test (5XSTS), Four Square Step Test (FSST), Berg Balance Scale (BBS), FGA, AMP, and the CHAMP. Data for performance-based measures were recorded on paper, later digitalized and stored in a REDCap database hosted at Northwestern University (8).

The 10MWT measures walking speed (9) by timing participants over the middle 10 meters of a 14-meter walk, with trials recorded at both a self-selected (SSV) and fast velocity (FV). The 6MWT assesses walking endurance (10) by measuring the distance participants cover within a 6-minute timeframe. The 5XSST provides a measure of lower extremity strength and transfer skill where the score is the time to stand and sit 5 times (11). The FSST measures functional mobility by timing participants as they step through a 2 × 2 grid on the floor, requiring changing step direction and increased foot clearance (12). The BBS quantifies static balance control and risk of falling through 14 balance tasks (13). The FGA assesses postural stability and balance during 10 walking tasks (14). The AMP measures the mobility potential of a person with LLA, and is often used to derive an appropriate K-Level (4). Participants perform static and dynamic sitting and standing activities, as well as transfer and gait skills of progressive difficulty. The CHAMP was designed to mitigate the ceiling effect of traditional outcome measures used for highly active individuals with LLA (15).

### Patient-reported outcome measures

The patient-reported outcome measures included the Orthotics and Prosthetics Users’ Survey (OPUS), Modified Falls Efficacy Scale (mFES), Prosthesis Evaluation Questionnaire (PEQ), Prosthetic Limb User Survey of Mobility (PLUS-M), five different PROMIS tests, Patient Health Questionnaire – 9 (PHQ-9), and Community Participation Indicators (CPI). Participants entered responses directly into validated digitalized forms in REDCap (16). Participants had the option to complete the electronic forms at home up to one week before the performance-based assessments. Those unable to fill out electronic forms were provided paper versions during the in-person visit, with researchers subsequently entering the data into REDCap.

The OPUS questionnaire assesses the effectiveness of orthotic and prosthetic devices in meeting user needs and their impact on quality of life (17).The mFES is a 14-item questionnaire that evaluates fear of falling by measuring an individual's perceived balance during daily activities (18). The PEQ evaluates a person's satisfaction with their prosthesis over various functional and social domains, such as ambulation and appearance (19, 20). The PLUS-M is a questionnaire that assesses prosthetic users’ perceived ability to perform indoor and outdoor activities that require use of both lower limbs (21). PROMIS, created by the National Institute of Health, are an expansive system of non-population-specific, person-centered measures for assessing physical, mental, and social health (22). The PHQ-9 is a 9-item questionnaire used to assess the presence and severity of depressive symptoms (23). The CPI is a measure of a person's satisfaction with their participation in the community (24).

For all outcome measures except 5XSTS, FSST, PROMIS Depression, and PHQ-9, a higher score is considered a better outcome.

### Data analysis

The primary objective of our analysis is to summarize reference data across various subgroups within the LLA population. Descriptive analyses detailed participant demographic characteristics and outcome measures, stratified by both amputation level and K-level. Participants with knee disarticulations were included with the TF group for the purposes of analysis.

## Results

Sixty-five participants enrolled in the larger clinical trial. Prior to the first assessment session, four participants withdrew from the study for health-related reasons and three due to conflicting time commitments. The remaining fifty-eight were included in this analysis (demographic information is summarized in Table 1). Overall, participants had a mean [range] age of 56 [24–76] years. Most were male (88%), had a K-Level of K3 (84%), and had unilateral amputations (93%).

**Table 1:** Participant demographics by amputation type and K-level. First section consists of unilateral users separated by K-level. Second section consists of bilateral and all users (bilateral and unilateral) separated by K level.

Amputation Type	Mean (SD) [range] or count (%)										
	Unilateral TT				Unilateral TF*				All Unilateral (TT and TF)		
*K-level*	*K2* *N = 1*	*K3* *N = 27*	*K4* *N = 4*	*All (K2-K4)* *N = 32*†	*K2* *N = 1*	*K3* *N = 19*	*K4* *N = 2*	*All (K2-K4)* *N = 22*†	*K3* *N = 46*	*K4* *N = 6*	*All (K2-K4)* *N = 54*†
Age (years)	63	59.6 (14.7) [28–76]	34.0 (18.7) [27–45]	56.5 (16.2) [27–76]	73	54.7 (16.5) [24–76]	41.5 (10.6) [34–49]	54.4 (16.5) [24–76]	57.6 (15.5) [24–76.0]	36.5 (8.9) [27–49]	55.6 (16.2) [24–76]
Weight (kg)^§^ ^(with prosthesis)^	118.0	94.0 (22.0) [43–148]	73.4 (17.4) [54–90]	92.1 =(22.4) [43–148]	90.7	94.1 (19.8) [63–133]	99.8 (9.6) [93–107]	94.4 (18.5) [63–133]	94.0 (20.9) [43–148]	82.2 (19.6) [54–107]	93.1 (20.8) [43–148]
Height (cm)	190.5	175.2 (7.4) [158–185]	174.9 (10.2) [165 –188]	175.6 (7.9) [157–191]	163.8	177.5 (9.6) [165 –192]	193.0 (3.6) [191–196]	178.3 (10.5) [164–196]	176.1 (8.3) [158–192]	181.0 (12.3) [165–196]	176.7 (9.1) [157–196]
Time since amputation (years)	2.1	14.2 (16.0) [0–55]	16.2 (14.0) [5–35]	14.1 (15.4) [0–55]	7.4	25.8 (21.3) [1–72]	1.2 (0.9) [1–2]	22.7 (21.3) [1–72]	19.0 (19.0) [0–72]	11.2 (13.3) [1–35]	17.6 (18.4) [0–72]
*Sex*											
Female	0 (0%)	5 (19%)	1 (25%)	6 (19%)	0	1 (5%)	0	1 (5%)	6 (13%)	*1 (17%)*	47 (87%)
Male	1 (100%)	22 (81%)	3 (75%)	26 (81%)	1 (100%)	18 (95%)	2 (100%)	21 (95%)	40 (87%)	*5 (83%)*	7 (13%)
*Cause of limb loss*											
Trauma	0	14 (52%)	4 (100%)	18 (56%)	0	11 (58%)	2 (100%)	13 (59%)	25 (51%)	6 (100%)	31 (57%)
Congenital	0	0	0	0	0	1 (5%)	0	1 (5%)	1 (2%)	0	1 (2%)
Vascular disease	1 (100%)	5 (19%)	0	6 (19%)	1 (100%)	2 (11%)	0	3 (14%)	7 (14%)	0	9 (17%)
Infection	0	5 (19%)	0	5 (16%)	0	1 (5%)	0	1 (5%)	6 (12%)	0	6 (11%)
Cancer	0	3 (11%)	0	3 (9%)	0	4 (21%)	0	4 (18%)	7 (14%)	0	7 (13%)
*Prescribed K-level*											
K2				1 (3%)				1 (5%)			2 (4%)
K3				27 (84%)				19 (86%)			46 (85%)
K4				4 (13%)				2 (9%)			6 (11%)
*Amputation Level*											
Unilateral TT									27 (59%)	4 (67%)	32 (59%)
Unilateral TF									19 (41%)	2 (33%)	22 (41%)
Bilateral TT/TT									N/A	N/A	N/A
Bilateral TT/TF									N/A	N/A	N/A
Bilateral TF/TF									N/A	N/A	N/A
*Military Status*											
Civilian	0	14 (52%)	4 (100%)	18 (56%)	0	12 (63%)	1 (50%)	13 (59%)	26 (57%)	5 (83%)	31 (57%)
Active Duty	0	1 (4%)	0	1 (3%)	0	1 (5%)	1 (50%)	2 (9%)	2 (4%)	01 (17%)	3 (6%)
Veteran	1 (100%)	12 (44%)	0	13 (41%)	1 (100%)	6 (32%)	0	7 (32%)	18 (39%)	0	20 (37%)
*Employment Status*											
Employed Full-Time	0	5 (19%)	2 (50%)	7 (22%)	0	8 (42%)	1 (50%)	9 (41%)	13 (28%)	3 (50%)	16 (30%)
Employed Part-Time	0	3 (11%)	0	3 (9%)	0	3 (16%)	0	3 (14%)	6 (13%)	0	6 (11%)
Retired	0	9 (33%)	0	9 (28%)	1 (100%)	5 (26%)	0	6 (27%)	14 (30%)	0	15 (28%)
On Disability Leave	1 (100%)	4 (15%)	0	5 (16%)		1 (5%)	1 (50%)	2 (9%)	5 (11%)	1 (17%)	7 (13%)
Other	0	5 (19%)	1 (25%)	6 (19%)	0	1 (5%)	0	1 (5%)	6 (13%)	1 (17%)	7 (13%)
No Response	0	1 (4%)	1 (25%)	2 (6%)	0	1 (5%)	0	1 (5%)	2 (4%)	1 (17%)	3 (6%)
*Type of Prosthetic Components*											
*Knee*	N/A	N/A	N/A	N/A							
MPK/Powered					1 (100%)	14 (74%)	2 (100%)	17 (77%)	See Unilateral TF (left)	See Unilateral TF (left)	See Unilateral TF (left)
Mechanical (hydraulic)					0	5 (26%)	0	5 (23%)			
*Foot*											
MADR/ESR‡	1 (100%)	22 (81%)	4 (100%)	27 (84%)	1 (100%)	19 (100%)	2 (100%)	22 (100%)	41 (89%)	6 (100%)	49 (91%)
Hydraulic	0	3 (11%)	0	3 (9%)	0	0	0	0	3 (7%)	0	3 (6%)
MPA	0	1 (4%)	0	1 (3%)	0	0	0	0	1 (2%)	0	1 (2%)
SACH	0	1 (4%)	0	1 (3%)	0	0	0	0	1 (2%)	0	1 (2%)
**Key** *N* = Sample size MPK, microprocessor-controlled knee; MADR, multi-axial dynamic response; ESR, energy storage and return; MPA, microprocessor-controlled ankle; SACH, solid ankle cushioned heel. *4 Participants had unilateral knee disarticulations. For purposes of reporting, these participants were grouped with the TF group, as they utilized a prosthetic knee component for function. ^†^There was only 1 K2 Participant within each amputation type (1 TT/ 1 TF/ 1 Bilateral) so they are not separated out under an individual K-Level, but they are included in the aggregate data. ^‡^Of those using a MADR or ESR foot, 3 (5.7%) had PIVOTs, 5 (9.4%) shock absorption, 4 (7.5%) torsion, and 1 (1.9%) adjustable height. ^§^ Weight values reflect measurements taken while wearing the prosthesis											

	Mean (SD) [range] or count (%)				
Amputation Type	Bilateral	All Types (Bilateral and Unilateral)			
*K-level*	*All (K2-K3)* *>N* *=* *4*†	*K2* *N* *=* *3*	*K3* *N* *=* *49*	*K4* *N* *=* *6*	*All (K2-K4)* *N* *=* *58*
Age (years)	60.5 (16.7) [36–72]	68.7 (5.13) [63–73]	57.6 (15.5) [24–76]	36.5 (8.9) [27–49]	56.0 (16.1) [24–76]
Weight (kg)	109.3 (25.4) [91–147]	104.0 (13.6) [91–118]	95.1 (21.6) [43–148]	82.2 (19.6) [54–107]	94.2 (21.3) [43–148]
Height (cm)	180.3 (9.0) [170–188]	176.5 (13.4) [164–191]	176.5 (8.5) [157–192]	181.0 (12.3) [165–196]	177.0 (9.0) [157–196]
Time since amputation (years)	27.9 (21.1) [6–52]	5.2 (2.7) [2–7]	20.0 (19.2) [0–72]	11.2 (13.3) [1–35]	18.3 (18.6) [0– 72]
*Sex*					
Female	0	*0*	*5 (19%)*	*1 (17%)*	7 (12%)
Male	4 (100%)	*3 (100%)*	*22 (81%)*	*5 (83%)*	51 (88%)
*Cause of limb loss*					
Trauma	2 (50%)	0	27 (55%)	6 (100%)	33 (57%)
Congenital	0	0	1 (2%)	0	1 (2%)
Vascular disease	2 (50%)	3 (100%)	8 (16%)	0	11 (19%)
Infection	0	0	6 (12%)	0	6 (10%)
Cancer	0	0	7 (14%)	0	7 (12%)
*Prescribed K-level*					
K2	1 (25%)				3 (5%)
K3	3 (75%)				49 (84%)
K4	0				6 (10%)
*Amputation Level*					
Unilateral TT	N/A	1 (33%)	27 (55%)	4 (67%)	32 (55%)
Unilateral TF	N/A	1 (33%)	19 (39%)	2 (33%)	22 (38%)
Bilateral TT/TT	2 (50%)	1 (33%)	1 (2%)	0	2 (3%)
Bilateral TT/TF	2 (50%)	0	2 (4%)	0	2 (3%)
Bilateral TF/TF	0	0	0	0	0
*Military Status*					
Civilian	0	0	26 (53%)	5 (83%)	31 (53%)
Active Duty	0	0	2 (4%)	1 (17%)	3 (5%)
Veteran	4 (100%)	3 (100%)	21 (43%)	0	24 (41%)
*Employment Status*					
Employed Full-Time	0	0	13 (27%)	3 (50%)	16 (28%)
Employed Part-Time	0	0	6 (12%)	0	6 (10%)
Retired	2 (50%)	2 (67%)	15 (31%)	0	17 (29%)
On Disability Leave	0	1 (33%)	5 (10%)	1 (17%)	7 (12%)
Other	1 (25%)	0	7 (14%)	1 (17%)	8 (14%)
No Response	1 (25%)	0	3 (6%)	1 (17%)	4 (7%)
*Type of Prosthetic Components*					
*Knee*					
MPK/Powered	2 (100%)	See Type of Prosthetic Components under Unilateral TF	See Type of Prosthetic Components under Unilateral TF and Bilateral	See Type of Prosthetic Components under Unilateral TF	See Type of Prosthetic Components under Unilateral TF and Bilateral
Mechanical (hydraulic)	0				
*Foot*					
MADR/ESR‡	4 (100%)	3 (100%)	44 (90%)	6 (100%)	53 (91%)
Hydraulic	0	0	3 (6%)	0	3 (5%)
MPA	0	0	1 (2%)	0	1 (2%)
SACH	0	0	1 (2%)	0	1 (2%)

§ Weight values reflect measurements taken while wearing the prosthesis.

Scores from the performance-based outcome measures are summarized in Table 2. Of note, 11 participants (19%) were not able to complete the 5XSTS test as they were unable to perform the test without using their arms. Two participants (3.4%) were unable to complete the FSST without touching a cane on the floor. Thirty-four participants (59%), all either K-level 3 or 4, performed the CHAMP. Scores from patient-reported outcome measures are summarized in Table 3. One participant did not complete any patient-reported measures due to time constraints.

**Table 2:** Performance-Based outcome measure scores. First section consists of unilateral users separated by K-level. Second section consists of bilateral and all users (bilateral and unilateral) separated by K level.

	Mean (SD) [range]										
Amputation Level	Unilateral TT				Unilateral TF				All Unilateral (TT and TF)		
*K-level* *N unless otherwise noted within the cell*	*K2* *N* *=* *1*	*K3* *N* *=* *27*	*K4* *N* *=* *4*	All (K2-K4)* N = 32	*K2* *N* *=* *1*	*K3* *N* *=* *19*	*K4* *N* *=* *2*	All (K2-K4)* N *=* *22*	*K3* *N* *=* *46*	*K4* *N* *=* *6*	All (K2-K4)* N = 54
10MWT – SSV (m/s)	0.98	1.20 (0.22) [0.70–1.70]	1.25 (0.16) [1.03–1.41}	1.20 (0.22) [0.70–1.70]	0.61	1.06 (0.21) [0.67–1.49]	1.25 (0.10) [1.19–1.32]	1.06 (0.22) [0.61–1.49]	1.14 (0.23) [0.67–1.70]	1.25 (0.13) [1.03–1.41]	1.14 (0.23) [0.61–1.70]
10MWT – FV (m/s)	1.42	1.61 (0.33) [0.98–2.26]	1.81 (0.27) [1.46–2.08]	1.63 (0.32) [0.98–2.26]	0.51	1.38 (0.37) [0.86–2.16]	1.67 (0.08) [1.61–1.73]	1.37 (0.40) [0.51–2.16]	1.52 (0.36) [0.86–2.26]	1.76 (0.22) [1.46–2.08]	1.52 (0.38) [0.51–2.26]
6MWT (m)	371.5	430.0 (110.5) [206.8 – 600.0] *N*=26‡	566.6 (91.8) [438.6–648.9]	445.8 (115.6) [206.8–648.9] *N* = 31‡	59.5	382.0 (128.8) [105.5–600.1]	499.0 (N/A) [N/A] *N* = 1§	372.2 (143.9) [59.5–600.1] *N* = 21§	409.8 (119.6) [105.5–600.1] *N* = 45‡	553.1 (85.0) [438.6–648.9] *N* = 5§	416.1 (131.6) [59.5–648.9] *N* = 52‡§
5XSTS (sec)	30 1 (100%)	13.8 (4.8) [7.2–27.5]	10.7 (1.9) [8.8–13.2]	13.9 (5.5) [7.2–30.0]	25.02 1 (100%)	18.9 (5.9) [9.7–29.8]	14.5 (0.1) [14.4–14.6]	18.5 (5.6) [9.7–29.8]	15.4 (5.7) [7.2–29.8]	12.0 (2.4) [8.8–14.6]	15.4 (5.9) [7.2–30.0]
N (%) Successful†		25 (93%)	4 (100%)	30 (94%)		12 (63%)	2 (100%)	15 (68%)	37 (80%)	6 (100%)	45 (83%)
FSST (sec)	15 1 (100%)	11.7 (5.3) [4.8–33.3]	9.2 (3.7) [5.9–12.8]	11.5 (5.1) [4.8–33.3]	52.39 1 (100%)	14.6 (7.9) [6.8–42.0]	11.2 (0.5) [10.8–11.5]	16.1 (11.1) [6.8–52.4]	12.9 (6.6) [4.8–42.0]	9.8 (3.1) [5.6–12.8]	13.3 (8.3) [4.8–52.4]
N (%) Successful†		26 (96%)	4 (100%)	31 (97%)		18 (95%)	2 (100%)	21 (95%)	44 (96%)	6 (100%)	52 (96%)
BBS score	50	49.0 (6.4) [26–56]	54.0 (1.6) [52–56]	49.6 (6.1) [26–56]	38	46.3 (8.0) [28–54]	55.5 (0.7) [55–56]	46.7 (8.1) [28–56]	47.9 (7.1) [26–56]	54.5 (1.5) [52–56]	48.4 (7.1) [26–56]
FGA score	19	22.9 (5.4) [10–30]	29.3 (1.0) [28–30]	23.6 (5.4) [10–30]	N/A *N* = 0||	18.7 (6.6) [9–28]	24.0 (2.83) [22–26]	19.2 (6.5) [9–28] *N* = 21||	21.2 (6.2) [9–30]	27.5 (3.1) [22–30]	21.9 (6.2) [9–30] *N* = 53||
AMP-Pro score	40	41.5 (4.6) [25–46]	45.8 (1.5) [44–47]	42.0 (4.5) [25–47]	37	37.8 (5.5) [23–44]	43.5 (0.7) [43–44]	38.3 (5.4) [23–44]	40.0 (5.3) [23–46]	45 (1.7) [43–47]	40.5 (5.2) [23–47]
CHAMP score	N/A	16.2 (8.1) [5.5–29.5]	25.1 (5.6) [17–29]	18.0 (8.4) [5.5–29.5]	N/A 0 (0%)	13.8 (7.1) [4–23]	13.5 (3.5) [11–16]	13.8 (6.6) [4–23]	15.2 (7.7) [4–29.5]	21.3 (7.6) [11–29]	16.3 (7.9) [4–29.5]
N (%) Attempted†		16 (59%)	4 (100%)	20 (63%)		11 (58%)	2 (100%)	13 (59%)	27 (59%)	6 (100%)	33 (61%)
**Key:** *N* = Sample size * There was only 1 K2 participant within each amputation type (1 TT/ 1 TF/ 1 Bilateral) so they are not separated out under an individual K-Level, but they are included in the aggregate data. ^†^ Some participants were unable to complete the 5XSTS and 4SST successfully, i.e., were unable to sit or stand without using their arms (5XSTS) or unable to complete the sequence without touching a cane on the floor (4SST). Similarly, only participants who were able to stand without assistance from a prone on the ground were asked to perform the CHAMP. The scores here are mean, SD, and range only of those who did complete the test. ^‡^ One K3 TT participant and one K3 bilateral participant did not complete the 6MWT test due to high blood pressure. ^§^ One K4 TF participant was unable to complete the 6MWT test due to his prosthetic knee overheating. ^||^ The K2 TF participant did not perform the FGA due to significant back pain.											

	Mean (SD) [range]				
Amputation Level	Bilateral	All Levels (i.e., TT, TF, and Bilateral)			
*K-level* *N unless otherwise noted within the cell*	All (K2-K3)* N *=* *4*	*K2* *N* *=* *3*	*K3* *N* *=* *49*	*K4* *N* *=* *6*	*All (K2-K4)* *N* *=* *58*
10MWT – SSV (m/s)	0.92 (0.17) [0.70–1.02]	0.76 (0.19) [0.61–0.98]	1.13 (0.22) [0.67–1.70]	1.25 (0.13) [1.03–1.41]	1.12 (0.23) [0.61–1.70]
10MWT – FV (m/s)	1.21 (0.25) [0.92–1.53]	0.95 (0.45) [0.51–1.42]	1.50 (0.36) [0.86–2.26]	1.76 (0.22) [1.46–2.08]	1.50 (0.38) [0.51–2.26]
6MWT (m)	317.1 (110.3) [193.2–404.6] *N* = 3‡	208.1 (156.5) [59.5–371.5]	408.5 (117.3) [105.5–600.1] *N* = 47‡	553.1 (85.0) [438.6–648.9] *N* = 5§	410.7 (131.6) [59.5–648.9] *N* = 55‡§
5XSTS (sec)	13.1 (0.1) [13.0–13.2]	27.5 (3.5) [25.0–30.0]	15.3 (5.6) [7.2–29.8]	12.0 (2.4) [8.8–14.6]	15.3 (5.8) [7.2–30.0]
N (%) Successful†	2 (50%)	2 (67%)	39 (80%)	6 (100%)	47 (81%)
FSST (sec)	18.2 (11.2) [8.0–33.0]	33.5 (18.7) [15.0–52.4]	12.9 (6.5) [4.8–42.0]	9.8 (3.1) [5.6–12.8]	13.7 (8.5) [4.8–52.4]
N (%) Successful†	4 (100%)	3 (100%)	47 (96%)	6 (100%)	56 (97%)
Berg score	38.5 (9.8) [25–47]	37.7 (12.5) [25–50]	47.6 (7.1) [26–56]	54.5 (1.5) [52–56]	47.8 (7.6) [25–56]
FGA score	15.0 (5.2) [9–21]	14.0 (7.1) [9–19] *N* = 2||	20.94 (6.1) [9–30]	27.5 (3.1) [22- 30]	21.4 (6.3) [9–30] *N* = 57||
AMPPRO score	34.8 (5.9) [28–41]	35.0 (6.2) [28–40]	39.8 (5.3) [23–46]	45.0 (1.7) [43–47]	40.1 (5.4) [23–47]
CHAMP score	N/A (N/A) [N/A]	N/A (N/A) [N/A]	15.1 (7.6) [4–29.5]	21.3 (7.6) [11–29]	16.2 (7.9) [4–29.5]
N (%) Attempted†	0 (0%)	0 (%)	28 (57%)	6 (%100)	34 (59%)

**Table 3:** Patient-Reported outcome measure scores. First section consists of unilateral users separated by K-level. Second section consists of bilateral and all users (bilateral and unilateral) separated by K level.

		Mean (SD) [range]									
Amputation Level		Unilateral TT				Unilateral TF			All Unilateral (TT and TF)		
*K-level* *N unless otherwise noted within the cell*		*K2* *N* *=* *1*	*K3* *N* *=* *27*	*K4* *N* *=* *4*	*All (K2-K4)* *N* *=* 32*	*K2* *N* *=* *1*	*K3* *N* *=* *19*	*All (K2-K4)* *N* *=* 21*†	*K3* *N* *=* *46*	*K4* *N* *=* *5*	*All (K2-K4)* *N* *=* 53*
**OPUS (Rasch score)**											
Health Quality of Life Index		56.1	60.3 (12.0) [42.7–100]	62.0 (9.8) [52.2–71.6]	60.4 (11.4) [42.7–100]	57.1	58.0 (5.5) [48.1–67.0]	58.0 (5.2) [48.1–67.0]	59.3 (9.8) [42.7–100]	61.2 (8.6) [52.2–71.6]	59.4 (9.5) [42.7–100]
Lower Extremity Functional Status		58.3	55.2 (10.2) [38.0 –90.8]	72.3 (18.7) [60.5–100]	57.5 (12.4) [38.0–100]	44.1	54.7 (8.5) [40.5–73.3]	54.6 (8.6) [40.5–73.3]	55.0 (9.4) [38.0–90.8]	70.4 (16.8) [60.5–100]	56.3 (11.1) [38.0–100]
Satisfaction with Device		45.7	50.7 (15.0) [32.0–100]	48.4 (5.4) [43.9–54.9]	50.3 (13.9) [32.0–100]	43.9	49.7 (11.5) [37.5–88.2]	49.7 (11.1) [37.5–88.2]	50.3 (13.5) [32.0–100]	49.7 (5.5) [43.9–54.9]	50.0 (12.7) [32.0–100]
**PEQ (score out of 100)**											
Ambulation		78.3	68.3 (23.9) [13.4–100]	86.7 (7.4) [80.6–97.5]	70.9 (22.9) [13.4–100]	37.6	69.5 (21.3) [17.6–94.7]	68.9 (21.8) [17.6–94.8]	68.8 22.6 [13.4–100]	86.9 (6.5) [80.6–97.5]	70.1 (22.3) [13.4–100]
Appearance		97.0	76.2 (20.1) [20.0–100]	69.0 (23.6) [45.0–99.0]	76.0 (20.3) [20.0–100]	88.3	70.8 (18.1) [25.0–94.8]	72.3 (17.9) [25–94.8]	74.0 (19.3) [20.0–100]	72.2 (21.6) [45.0–99.0]	74.5 (19.3) [20.0–100]
Frustration		69.1	73.0 (30.1) [0–100]	75.5 (20.5) [45.5–91.5]	73.2 (28.3) [0–100]	99.0	69.1 (28.7) [17.5–100]	71.5 (28.3) [17.5–100]	71.4 (29.2) [0–100]	78.2 (18.7) [45.5–91.5]	72.5 (28.0) [0–100]
Perceived Response		90.6	88.5 (15.0) [51.7–100] *N* = 24‡	91.0 (9.4) [84.3–97.6] *N* = 2‡	88.7 (14.2) [51.7–100] *N* = 27‡	96.6	91.8 (9.0) [75.2–100] *N* = 18‡	92.0 (8.6) [75.2–100] *N* = 20‡	89.9 (12.7) [51.7–100] *N* = 42‡	91.1 (6.6) [84.3–97.6] *N* = 3‡	90.1 (12.2) [51.7–100] *N* = 47‡
Residual Limb Health		20.4	73.4 (19.5) [2.3–100]	73.3 (10.8) [59.5–82.2]	71.7 (20.4) [2.3–100]	87.5	79.0 (10.8) [57.3–94.2]	80.0 (10.7) [57.3–94.2]	75.7 (16.5) [2.3–100]	76.7 (12.2) [59.5–90.7]	75.0 (17.6) [2.3–100]
Social Burdens		92.6	81.1 (21.6) [30.9–100] *N* = 16‡	83.8 (8.7) [77.7–90.0] *N* = 2‡	82.0 (20.0) [30.9–100] *N* = 19‡	56.0	83.8 (17.7) [43.5–100] *N* = 14‡	82.4 (18.0) [43.5–100] *N* = 16‡	78.1 (23.2) [17.0–100] *N* = 36‡	85.2 (6.6) [77.7–90] *N* = 3‡	82.2 (18.8) [30.9–100] *N* = 35‡
Sounds		25.0	70.5 (27.8) [0.5–100]	60.4 (41.3) [24.5–100]	67.8 (29.8) [0.5–100]	92.0	76.2 (22.6) [32.5–100]	78.1 (22.3) [32.5–100]	72.8 (25.7) [0.5–100]	68.1 (39.7) [24.5–100]	71.9 (27.3) [0.5–100]
Utility		29.4	74.1 (21.5) [9.1–97.1]	81.3 (12.6) [66.3–96.6]	73.6 (21.8) [9.1–97.1]	38.9	72.4 (17.5) [32.5–93.5]	71.5 (18.6) [32.5–93.5]	73.4 (19.8) [9.1–97.1]	82.7 (11.3) [66.3–96.6]	72.8 (20.4) [9.1–97.1]
Well Being		94.1	77.0 (21.5) [33.0–100]	89.3 (8.1) [78.5–96]	79.1 (20.5) [33.0–100]	63.0	74.0 (18.1) [35.0–100]	72.4 (18.1) [35.0–100]	75.8 (20.0) [33.0–100]	81.4 (18.9) [50.0–96.0]	76.4 (19.7) [33.0–100]
Mobility (Ambulation + Transfers)		65.4	71.4 (23.1) [12.3 –99.8]	89.4 (6.4) [84.7–98.3]	73.4 (22.2) [12.3–99.8]	43.3	70.8 (21.3) [18.1–93.3]	70.5 (21.6) [18.1–93.3]	71.1 (22.1) [12.3–99.8]	89.9 (5.6) [84.7–98.3]	72.2 (21.8) [12.3–99.8]
**mFES**											
Score		9.6	8.7 (1.4) [4.6–10]	9.2 (1.0) [7.9–10]	8.8 (1.4) [4.6–10]	7.1	8.4 (1.8) [4.4–10]	8.4 (1.8) [4.4–10]	8.6 (1.6) [4.4–10]	9.4 (0.9) [7.9–10]	8.7 (1.6) [4.4–10]
**PLUS-M**											
T-score		52.7	53.3 (7.3) [39.0–71.4]	58.6 (7.4) [49.1–67.1]	53.9 (7.3) [39.0–71.4]	41.5	52.3 (6.9) [34.1–62.5]	52.1 (7.2) [34.1–62.5]	52.9 (7.1) [34.1–71.4]	58.8 (6.4) [49.1–67.1]	53.2 (7.2) [34.1–71.4]
**PROMIS (T-score)**											
Physical Function for Mobility Aid Users		39.4	43.1 (6.7) [33.9–67.4]	51.5 (8.3) [41.1–60.8]	44.0 (7.3) [33.9–67.4]	32.3	42.9 (6.1) [31.8–56.2]	42.6 (6.3) [31.8–56.2]	43.0 (6.4) [31.8–67.4]	50.7 (7.3) [41.1–60.8]	43.5 (6.9) [31.8–67.4]
Mobility		43.4	43.1 (5.3) [31.9–60.2]	51.4 (7.4) [42.8–60.2]	44.2 (6.1) [31.9–60.2]	35.0	41.8 (4.9) [31.6–49.2]	41.8 (5.1) [31.6–49.2]	42.6 (5.1) [31.6–60.2]	50.7 (6.6) [42.8–60.2}	43.2 (5.8) [31.6–60.2]
Depression		56.8	46.4 (10.0) [38.2–64.8]	51.4 (9.1) [38.2–59.1]	47.4 (9.9) [38.2–64.8]	54.2	49.3 (7.2) [38.2–62.1]	49.7 (7.0) [38.2–62.1]	47.6 (9.0) [38.2–64.8]	51.6 (7.9) [38.2–59.1]	48.3 (8.9) [38.2–64.8]
Satisfaction with SRA		35.2	52.6 (8.0) [36.8–65.6]	56.5 (9.1) [44.0–65.6]	52.5 (8.6) [35.2–65.6]	40.3	53.0 (7.1) [41.7–65.6]	52.0 (7.4) [40.3–65.6]	52.7 (7.5) [36.8–65.6]	54.3 (9.4) [44.0–65.6]	52.3 (8.1) [35.2–65.6]
Ability to Participate in SRA		49.9	50.3 (8.5) [34.7–65.4]	51.6 (10.2) [41.0–65.4]	50.5 (8.4) [34.7–65.4]	37.6	50.7 (5.8) [43.0–65.4]	49.8 (6.3) [37.6–65.4]	50.5 (7.4) [34.7–65.4]	50.0 (9.5) [41.0–65.4]	50.2 (7.6) [34.7–65.4]
**PHQ-9**											
Score		8	4.3 (6.2) [0–24]	5.0 (4.2) [0–9]	4.5 (5.8) [0–24]	N/A	4 (3.9) [0–15]	4.1 (3.8) [0–15] *N* = 20§	4.2 (5.3) [0–24]	5.2 (3.7) [0–9]	4.3 (5.1) [0–24] *N* = 52§
Depression Severity	None/Minimal		16 (59.3%)	2 (50%)	18 (56.3%)		13 (68.4%)	13 (65%)	29 (63%)	2 (40%)	31 (59.6%)
	Mild		8 (29.6%)	2 (50%)	11 (34.4%)		5 (26.3%)	6 (30%)	13 (28.3%)	3 (60%)	17 (32.7%)
	Moderate		0 (0%)	0 (0%)	0 (0%)		0 (0%)	0 (0%)	0 (0%)	0 (0%)	0 (0%)
	Moderately-Severe		2 (7.4%)	0 (0%)	2 (6.3%)		1 (5.3%)	1 (5%)	3 (6.5%)	0 (0%)	3 (5.8%)
	Severe		1 (3.7%)	0 (0%)	1 (3.1%)		0 (0%)	0 (0%)	1 (2.2%)	0 (0%)	1 (1.9%)
**CPI**											
Important Activities Done Often Enough (%)		75.0	78.1 (21.3) [17.6–100]	53.9 (32.6) [25–94.1]	75.0 (23.5) [17.6–100]	25.0	65.6 (26.1) [18.8–100]	64.9 (26.9) [18.8–100]	73.0 (23.9) [17.6–100]	61.3 (32.7) [25.0–94.1]	71.0 (25.1) [17.6–100]
Involvement in Life Situations (Rasch score)		58.2	53.7 (6.9) [43.1–67.1]	56.9 (10.4) [45.4–70.3]	54.3 (7.2) [43.1–70.3]	51.5	57.1 (13.0) [43.1–100]	56.7 (12.4) [43.1–100]	55.1 (9.9) [43.1–100]	56.6 (9.0) [45.4–70.3]	55.2 (9.6) [43.1–100]
Control over Participation (Rasch score)		63.6	65.9 (11.3) [48.4–100]	62.3 (8.6) [54.8–74.2]	65.4 (10.7) [48.4–100]	65.0	66.8 (15.5) [49.3–100]	66.7 (14.7) [49.3–100]	66.3 (13.0) [48.4–100]	63.1 (7.6) [54.8–74.2]	65.9 (12.3) [48.4–100]
**Key:** *N* = sample size * There was only 1 K2 Participant within each amputation type (1 TT/ 1 TF/ 1 Bilateral) so they are not separated out under an individual K-Level, but they are included in the aggregate data. ^†^ There was only 1 K4 TF participant who completed patient-reported outcome measures, so that data is not separated out under an individual K-Level, but it is included in the aggregate data. The other K4 TF did not complete any patient-reported outcome measures due to time constraints. ^‡^ Six and 18 participants overall had invalid scores for the Perceived Response (5 items) and Social Burden (3 items) subscales, respectively; i.e., more than half of the subscale's questions (rounding up if odd) were scored as “no response”. ^§^ One K2 TF amputee mistakenly did not complete the PHQ-9.											

		Mean (SD) [range]				
Amputation Level		Bilateral	All Levels (i.e., TT, TF, and Bilateral)			
*K-level* *N unless otherwise noted*		*All (K2-K3)* *N* *=* 4*	*K2* *N* *=* *3*	*K3* *N* *=* *49*	*K4* *N* *=* *5*	*All (K2-K4)* *N* *=* *57*
**OPUS (Rasch score)**						
Health Quality of Life Index		58.0 (3.0) [53.9–60.9]	57.5 (1.6) [56.1–59.2]	59.2 (9.5) [42.7–100]	61.2 (8.6) [52.2–71.6]	59.3 (9.2) [42.7–100]
Lower Extremity Functional Status		53.8 (6.4) [48.2–61.3]	50.4 (7.2) [44.1–58.3]	55.0 (9.2) [38.0–90.8]	70.4 (16.8) [60.5–100]	56.1 (10.8) [38.0–100]
Satisfaction with Device		45.8 (3.1) [41.5–48.5]	46.0 (2.3) [43.9–48.5]	50.0 (13.2) [32.0–100]	50.0 (5.5) [43.9–54.9]	49.7 (12.3) [32.0–100]
**PEQ (score out of 100)**						
Ambulation		59.3 (27.7) [34.3–83.5]	50.7 (23.9) [36.3–78.3]	68.7 (22.7) [13.4–100]	86.9 (6.5) [80.6–97.5]	69.3 (22.6) [13.4–100]
Appearance		74.8 (23.0) [54.2–95.1]	93.2 (4.5) [88.3–97.0]	73.6 (19.3) [20.0–100]	72.2 (21.6) [45.0–99.0]	74.5 (19.3) [20.0–100]
Frustration		79.6 (34.5) [28.0–99.4]	88.8 (17.1) [69.1–99.0]	71.5 (29.4) [0–100]	78.2 (18.7) [45.5–91.5]	73.0 (28.2) [0–100]
Perceived Response		84.1 (21.5) [51.7–99.3]	94.6 (3.5) [90.6–96.6]	89.2 (13.6) [51.7–100] *N* = 45‡	91.1 (6.6) [84.3–97.6] *N* = 3‡	89.6 (12.9) [51.7–100] *N* = 51‡
Residual Limb Health		76.1 (5.9) [71.9–84.6]	59.9 (35.1) [20.4–87.5]	75.8 (16.1) [2.3–100]	76.7 (12.2) [59.5–90.7]	75.1 (17.0) [2.3–100]
Social Burdens		79.3 (16.7) [61.6–99.4]	70.1 (19.7) [56.0–92.6]	82.6 (19.0) [30.9–100] *N* = 33‡	85.2 (6.6) [77.7–90.0] *N* = 3‡	81.9 (18.4) [30.9–100] *N* = 39‡
Sounds		54.3 (12.8) [39.0–67.5]	59.6 (33.6) [25.0–92.0]	71.6 (25.6) [0.5–100]	68.1 (39.7) [24.5–100]	70.6 (26.8) [0.5–100]
Utility		70.2 (12.3) [53.1–82.3]	47.5 (23.7) [29.4–74.4]	73.1 (19.4) [9.1–97.1]	82.7 (11.3) [66.3–96.6]	72.6 (19.9) [9.1–97.1]
Well Being		73.2 (27.5) [33.5–95.5]	78.2 (15.6) [63.0–94.1]	75.5 (20.6) [33.0–100]	81.4 (18.9) [50.0–96.0]	76.2 (20.0) [33.0–100]
Mobility (Ambulation + Transfers)		66.0 (24.3) [40.0–88.5]	53.1 (11.3) [43.3–65.4]	71.1 (22.1) [12.3–99.8]	89.9 (5.6) [84.7–98.3]	71.8 (21.8) [12.3–99.8]
**mFES**						
Score		7.7 (2.5) [5.2–9.9]	7.3 (2.2) [5.2–9.6]	8.6 (1.6) [4.4–10]	9.4 (0.9) [7.9–10]	8.6 (1.6) [4.4–10]
**PLUS-M**						
T-score		48.3 (6.2) [40.3–53.6]	44.8 (6.8) [40.3–52.7]	52.8 (6.9) [34.1–71.4]	58.8 (6.4) [49.1–67.1]	52.9 (7.2) [34.1–71.4]
**PROMIS (T-score)**						
Physical Function for Mobility Aid Users		38.2 (5.7) [31.7–44.3]	34.5 (4.3) [31.7–39.4]	42.8 (6.3) [31.8–67.4]	50.7 (7.3) [41.1–60.8]	43.1 (6.9) [31.7–67.4]
Mobility		39.5 (1.6) [37.7–41.4]	39.0 (4.2) [35.0–43.4]	42.4 (5.0) [31.6–60.2]	50.7 (6.6) [42.8–60.2]	43.0 (5.7) [31.6–60.2]
Depression		48.7 (8.5) [38.2–58.3]	52.4 (5.5) [46.3–56.8]	47.7 (9.0) [38.2–64.8]	51.6 (7.9) [38.2–59.1]	48.3 (8.8) [38.2–64.8]
Satisfaction with SRA		52.1 (10.2) [43.1–65.6]	39.5 (4.0) [35.2–43.1]	52.9 (7.6) [36.8–65.6]	54.3 (9.4) [44.0–65.6]	52.3 (8.1) [35.2–65.6]
Ability to Participate in SRA		46.1 (5.1) [38.7–49.9]	42.1 (6.8) [37.6–49.9]	50.4 (7.2) [34.7–65.4]	50.0 (9.5) [41.0–65.4]	49.9 (7.5) [34.7–65.4]
**PHQ-9**						
Score		3.8 (2.2) [2–7]	5.5 (3.5) [3–8] *N* = 2§	4.2 (5.1) [0–24]	5.2 (3.7) [0–9]	4.3 (4.9) [0–24] *N* = 56§
Depression Severity	None/Minimal	3 (75%)	1 (50%)	31 (63.3%)	34 (61%)	34 (61%)
	Mild	1 (25%)	1 (50%)	14 (28.6%)	18 (32%)	18 (32%)
	Moderate	0 (0%)	0 (0%)	0 (0%)	0 (0%)	0 (0%)
	Moderately-Severe	0 (0%)	0 (0%)	3 (6.1%)	3 (5%)	3 (5%)
	Severe	0 (0%)	0 (0%)	1 (2.0%)	1 (2%)	1 (2%)
**CPI**						
Important Activities Done Often Enough (%)		76.7 (17.6) [60.0–100]	53.3 (25.7) [25.0–75.0]	73.5 (23.5) [17.6–100]	61.3 (32.7) [25.0–94.1]	71.4 (24.6) [17.6–100]
Involvement in Life Situations (Rasch score)		51.8 (12.8) [41.5–70.3]	50.4 (8.4) [41.5–58.2]	55.1 (9.9) [43.1–100]	56.6 (9.0) [45.4–70.3]	55.0 (9.7) [41.5–100]
Control over Participation (Rasch score)		64.9 (8.4) [53.8–74.2]	64.5 (0.8) [63.6–65.0]	66.2 (12.8) [48.4–100]	63.1 (7.6) [54.8–74.2]	65.9 (12.0) [48.4–100]

Complete data for each participant and outcome measure are available in Supplementary Material 2, which also provides detailed information on assistive device use, level of assistance, prosthetic foot type, and when applicable knee component type, as well as other specific testing conditions.

## Discussion

This study enriches existing literature by providing population-specific reference values for outcome measures used in the LLA population, together with clear documentation of the administration procedures applied across sites. By reporting scores stratified by K-level for each amputation level and in aggregated groups, this study allows clinicians and researchers to interpret outcomes within the context of comparable patient profiles, supporting goal setting, progress monitoring, and communication with payers, while avoiding inappropriate comparisons with general population norms. Reporting individual-level scores and demographic characteristics further promotes transparency and facilitates future cross-study comparisons.

To the best of our knowledge, this study is the first to report scores for the LLA population on the PROMIS Physical Function for Mobility Aid Users and Mobility scales, and the CPI Percentage of Important Activities Done Often Enough. Apart from Erbes et al. (25) (*N* = 235, Veterans, mean age 64 years), who had a notably different cohort and scored differently on all common measures, we are also the first to report scores for the PROMIS Ability to Participate in SRA and CPI Involvement in Life Situations and Control over Participation scales for an LLA population.

The average scores found are similar to those of existing literature for the following outcome measures used with an LLA population: FGA (26); AMP (4, 5, 27); PEQ: Ambulation, Appearance, Frustration, Residual Limb Health, and Utility (27); PHQ-9 (28, 29); and again excluding Erbes et al., PROMIS Depression and Satisfaction with SRA scales (30).

For the remaining outcomes, deviations from previous reports can likely be explained by differences in cohort composition or testing protocol, as outlined below. In all but a few cases, scores exhibited expected trends for functional capacity of various groups (e.g., those designated K4 outperformed K3; the TT group outperformed the TF group).

### 10MWT

For each K-level, 10MWT speeds, both SSV and FV, are faster than those previously reported by Sions et al. (*N* = 55) and Beisheim et al. (*N* = 41) (31, 32). A critical methodological difference could account for the lower speeds of the other studies, across all groups: calculating the speed by first averaging the 3 trial times then dividing by the distance—rather than calculating the speed of each trial then averaging—would underestimate the actual average speed. Additionally, both of these studies used a shorter track: a 6 m timed track segment compared to our 10 m. Though both versions excluded the acceleration and deceleration phases of the walk, the difference in track length might still affect the calculated speed. Finally, in both prior studies consisted of a substantially larger proportion of participants with vascular-disease etiology (58% and 40%) compared to ours (17%). Individuals with LLA from vascular-disease etiologies have consistently been shown to walk slower than those with other etiologies (33, 34). On the other hand, our walking speeds align closely with the self-selected speeds reported by Akarsu et al. (35) for individuals with both unilateral and bilateral LLA, and with those calculated for all unilateral sub-groups from Sawers et al. (36). However it is unreported whether Akarsu et al. timed the acceleration and deceleration stages of the walk. This is the first study to report scores categorized by K-level for both amputation levels (TT and TF) at both assessed speeds and with a 10 m timed length.

### 6MWT

In our study, the mean 6MWT scores are markedly higher than those reported by Sions et al. (*N* = 35 K3 and *N* = 20 K4) (31). As their participants were also slower walkers than ours on the 10MWT, we expect this is a difference of the makeup of the cohorts. Similarly, our scores were appreciably higher than those reported by Resnik et al. (*N* = 44, unilateral) (27). A plausible explanation is the 10-year higher mean age of their participants. Gailey et al. report 6MWT scores by K-level for 167 LLA but used a different protocol: where other studies instructed participants to “cover as much ground as possible”, Gailey et al. instructed participants to walk at a comfortable speed (4). In fact, their participants walked a noticeably shorter distance on average across all groups. Our study is the first to report 6MWT scores by both amputation level and K-level, with the directive to cover the greatest distance.

### 5XSTS and FSST

Two previous studies report 5XSTS and FSST scores for an LLA cohort (37, 38). Beisheim et al. (*N* = 67) observed considerably better scores (lower completion times) for the overall group compared to those in our study (37). For the 5XSTS, this can be attributed to a shorter portion of the test being timed. Specifically, they stopped timing at the peak of the 5th stand while we stopped when the participant returned to a seated position. For the FSST, Beisheim et al. used tape on the floor instead of canes which likely facilitated the task by requiring less effort and time to lift feet over obstacles. Wilken et al. (*N* = 19) have significantly better scores on both tests, likely because their participants were all active-duty military personnel, who are presumably more functional on average than those in our study (38). Considering differences in protocol and cohort composition, the observed trends in scores are reasonable, and align with expected functional differences among various groups. The high failure rate in the 5XSTS should be considered before selecting it as an evaluation tool for LLA.

### BBS

Previous research has often aggregated BBS scores for individuals with unilateral TT and TF LLA (39), or for unilateral and bilateral LLA (40), stratified them by various balance abilities (41, 42), or included only one amputation level (41–43). In our study, we observed notable differences between unilateral K3 and K4 participants and between unilateral and bilateral participants, indicating the utility in reporting scores by these group (though no distinct difference was found between TT and TF participants).

### CHAMP

Our CHAMP results were markedly lower than those reported by Gailey et al. for each TT, TF, and combined unilateral amputations (6). This difference is likely again in the respective demographics of the cohorts: their participants were all active duty or retired service men with a mean age of 29 ± 6 years, where our participants were primarily non-military with a mean age of 56 ± 16 years. Of note, Gailey et al. required participants to have minimum AMP and 6MWT scores in order to participate in the CHAMP, while we only required that participants be able to safely stand from prone on the ground. However, we expect the difference in protocol to have had little effect, as scores in our study were comparable to those of two other studies (7, 44) who used similar protocols to Gailey, but with cohorts more similar to ours (non-military, average age >38 years).

### OPUS

Resnik et al. reported scores for the same components of the OPUS without distinguishing between K-level or type of amputation (27). Their scores for the Health-related Quality of Life and for Lower Extremity Functional Status components were notably lower compared to those in the current study, while their Satisfaction with Device scores were not. A plausible explanation for this discrepancy is the update to the OPUS scoring guide, which included minor modifications to the Rasch code tables. For this reason, raw scores are provided in Supplementary Material 2.

### mFES

Barnett et al. represents the only other paper, to our knowledge, that explored the mFES with the LLA population (45). Their research reported mFES scores exclusively for 7 unilateral TT males 1 to 6 months post-discharge from rehabilitation. Considering our TT participants have substantially more experience with prostheses (13 years more, on average), it is understandable that participants in our study reported markedly higher confidence in balance than those in Barnett's study.

### PEQ

PEQ scores for Perceived Response, Social Burden, and Well-Being, were notably different than those of Resnik et al. (27). As with the 6MWT, the difference with scores from Resnik et al. is likely explained by the difference in average age of the two study groups. Our scores for the Mobility subscale were markedly higher than those reported by Franchignoni et al. (46). First, our participants had a longer mean time since amputation (18.3 years), allowing for better long-term adaptation to prosthetic use, compared to the shorter adaptation period in Franchignoni's (up to 5 years) study. Second, Hafner's study had a higher percentage of participants with vascular-disease etiologies (43.7%) compared to ours (17%), who may struggle with the mobility tasks assessed more than those with other etiologies. These factors might explain why our cohort demonstrated higher mobility scores.

### PLUS-M

While our participants’ PLUS-M T-scores are higher on average than those of Hafner et al. (5) and Erbes et al. (25), they are similar to those of the development sample (21). Additionally, Erbes's cohort had a mean age 10 years greater than ours, aligning with the inverse relationship between age and score exhibited in the development sample.

### Study limitations

While this report provides score summaries by K-level, the majority of participants enrolled in this study had unilateral amputation classified at a K3 level (only 3 K2-level, 6 K4-level, and 4 bilateral participants were enrolled), which limit the utility of this dataset for those specific sub-categories. In addition, the sample included a predominance of male participants, reflecting typical enrollment patterns in prosthetic rehabilitation studies, particularly that include military and veteran populations. This imbalance limits the ability to explore potential sex-specific differences in performance and patient-reported outcomes, despite evidence that such differences exist in body composition, strength, gait biomechanics, and health and quality-of-life measures between males and females (47–49) As such, reference values for less-represented groups (e.g., K2, K4, bilateral amputees and female) should be interpreted cautiously, used in conjunction with individual patient factor and considered as one component of a broader clinical assessment rather than as standalone indicators of performance or recovery. Future work should aim to recruit a more balanced cohort across sex and functional levels to confirm reference ranges, explore potential sex-specific patterns, and establish meaningful clinically important difference metrics for these outcome measures.

## Conclusions

This study provides clear documentation of the administration procedures used for each outcome measure and presents population-specific reference values stratified by amputation levels and K-levels, aiming to clearly articulate where our findings align or diverge from existing literature. We explore potential reasons for any discrepancies, including variations in test execution, scoring, and participant characteristics. This work also highlights areas where LLA-specific normative data remain limited and, while not intended to establish formal norms, the present findings offer preliminary reference values that can aid interpretation, support clinical benchmarking, and inform future research and rehabilitation planning. Building on this foundation, future research should incorporate longitudinal study designs to characterize changes in outcomes and to establish empirically based criteria for clinically meaningful change over time.

## Data Availability

The original contributions presented in the study are included in the article/Supplementary Material, further inquiries can be directed to the corresponding authors.
