# Supplementary material for: References values and standardized testing protocols for performance-based and patient-reported outcome measures among individuals with lower limb amputation
Source: Front Rehabil Sci. 2026 Mar 13;7:1786298. doi: 10.3389/fresc.2026.1786298 (PMC13021846; doi:10.3389/fresc.2026.1786298)
Supplement: Supplementary file 1 [file Supplementaryfile1.docx]

**Appendix 1: Assessment Protocols**

This Appendix details the protocols used in this study for each assessment. Unless otherwise noted, assistive devices (ADs) were allowed as needed for safety. Equations used to compare scores with other scales from previous literature are also provided. Where applicable, relevant pdf forms and scoring guides are provided in the **Supplementary Material 1**. The REDCap surveys of the forms digitized for this study are provided in **Supplementary Material 2**.

*10 Meter Walk Test (10MWT) Self-Selected (SSV) and Fast (FV) velocities*

Participants walk 14 meters across a flat, indoor surface. The middle 10 meters of the walkway are timed, to allow for a 2-meter acceleration in the beginning, and a 2-meter deceleration at the end. Participants perform 3 trials of the test at their own self-selected pace, and 3 trials at their fastest safe pace. The speed of each trial is calculated and then averaged for both the self-selected and fast paces. Special care was taken to first calculate the speed of each and then average the speeds (Equation 1, left side), as opposed to averaging the trial times and then calculating the speed (Equation 1, right side). The error of the latter becomes minimal and easily overlooked when trial times are similar, which is typically the case.

**Equation 1: Average 10MWT speed calculation method inequality**

$$\frac{1}{3}\left( \frac{10 m}{t_{1}}+\frac{10 m}{t_{2}}+ \frac{10 m}{t_{3}} \right)\neq\frac{10 m}{\frac{1}{3}\left( t_{1}+t_{2}+t_{3} \right)}$$

$${where t}_{1},t_{2}, {and t}_{3} are the three trial times$$

*6 Minute Walk Test (6MWT)*

Participants walk a pre-determined course on a flat, indoor surface with minimal turns, aiming to maximize the distance covered within six-minute timeframe. The score is the total distance covered. While standing rest breaks are acceptable, the test ends if a participant requires a seated break, with their score being the distance they cover up to that point. Of note, for this study, participants at two of the study sites walk back and forth on a straight 30.48-meter (100-foot) walkway making a 180 degree turn at each end. Given space constraints, participants at the third site walked a 54-meter (177.2-foot) hallway then continued on a 57-meter (187-foot) looped path.

*Five Times Sit to Stand Test (5XSST)*

Participants begin seated in a standard-height unsecured chair (45-50 cm from seat to floor), with their arms folded across their chest. They are instructed to stand up fully and then sit back down five times, as quickly yet safely as possible. The time taken to complete the test is the score. If a participant uses their arms or an AD for support, the test is considered a failure. Each participant is allowed only one timed attempt, though a practice trial is allowed to demonstrate understanding of the movement pattern.

*Four Square Step Test (FSST)*

The FSST involves a 2x2 grid created by arranging four canes in a cross-pattern on the floor, delineating four squares. Starting in one square, participants step sequentially into each square—forward, right, backward, left—then reverse the sequence back to the starting square, remaining front-facing throughout. A diagram is provided in **Supplementary Material 1** page 1 for visualization. Success requires that both feet contact the ground within each square without touching the canes. Timing starts when the first foot contacts the floor with the first forward step and ends when the last foot touches the floor upon return to the starting square. Participants are instructed to move as quickly yet safely as possible. A practice trial is allowed for movement familiarization. Participants then complete two trials, and the time of the fastest trial is the score.

*Berg Balance Scale (BBS)*

For the BBS, participants perform a series of 14 different activities meant to challenge balance in different situations. See pages 2-5 of **Supplementary Material 1** for the activity and scoring details. Activities include standing with various bases of support, sitting, transfers, reaching, standing with eyes closed, performing weight shifts/turns, and tapping steps. Each item is scored on a 0-4 scale, based on ability to perform the activity. The final score is the sum of the individual scores, with a higher score representing better balance control. ADs are not allowed for task completion.

*Functional Gate Assessment (FGA)*

The FGA requires participants to perform a series of 10 different walking activities over a 6-meter walking area. See pages 6-9 of **Supplementary Material 1** for the activity and scoring details. Activities include walking at varying speeds, walking with head turns, performing body turns, stepping over obstacles, walking with a narrow base of support, walking with eyes closed, retro walking, and navigating stairs. Each item is scored on a 0-3 scale, based on ability to perform the activity. The final score is the sum of the individual scores, with a higher score representing better stability. Use of an AD is allowed but influences the final score.

*Amputee Mobility Predictor Assessment Tool (AMP)*

The AMP measures the mobility potential of a person with LLA, either with a prosthesis (AMP-Pro) or without (AMP-NoPro). The current study only tested the AMP-Pro. It is used to predict functional mobility and often to derive an appropriate K-Level^1^. Participants perform static and dynamic sitting and standing activities, as well as transfer and gait skills of progressive difficulty. Scores are delineated for each activity and summed for a final score. See pages 10-11 of **Supplementary Material 1** for the activity and scoring details. Use of an AD is allowed but influences the final score.

*Comprehensive High-Level Activity Mobility Predictor (CHAMP)*

The CHAMP consists of 4 tests with one final score: Single-leg stance with both lower extremities, the Edgren sidestep test, the T-Test, and the Illinois Agility Test^2^. We used the instructions and scoring guides provided in Appendices G and J, respectively, of Gaunaurd 2012^3^, excluding the “Medicine Ball Put” test. These are provided in **Supplementary Material 1** (pp. 12-17) for convenience. In contrast to previous research^2,4–6^, the current study had no baseline requirements such as the ability to jog and run, walk unaided, achieve a minimum distance with a walking test or reach a specific AMP score to perform the CHAMP. However, participants who were unable or unwilling to stand unassisted from the floor from a prone position—a prerequisite for the Illinois Agility Test—did not perform the assessment.

*Orthotics and Prosthetics Users’ Survey (OPUS)*

The OPUS is a self-report questionnaire for orthosis and prosthesis users. In addition to a component for individuals with upper limb amputation, the OPUS includes three components for lower-limb prosthesis users: Health Related Quality of Life Index (23 items), Lower Extremity Functional Status (20 items), and Satisfaction with Device and Services (11 device-related, 10 service-related items. Though participants answered all questions in the latter, we only considered the device-related items, as we were providing no services. Respondents choose from five options on Likert scale for each items, and scores for each component are calculated by summing responses according to the scoring guide given by Heinemann et al^7–9^. The scoring guide also provides a conversion to Rasch Measure (0-100 scale), which is utilized in our study. For all components a higher score indicates a better outcome. Forms and the scoring guide are provided in **Supplementary Material 1** (pp. 18-28).

*Prosthesis Evaluation Questionnaire (PEQ)*

The PEQ consists of 9 functional validated domain scales: Ambulation, Appearance, Frustration, Perceived Response, Residual Limb Health, Social Burden, Sounds, Utility, and Well-Being^10^. An additional scale of Mobility may be created by combining ambulation and transfer questions, as suggested by Miller et al^11^. Though some adaptations of the PEQ employ various Likert-scale responses ^12–14^, our study used the original analog format, adapted into a slider on a response bar for the electronic version, with scores ranging from 0 to 100^10^.

When comparing to studies that used a 0-7 point Likert scale^15^, we normalized our scores using the equation **Score_7-pt_ = (7 x Score_100-pt_) / 100**. Similarly, when comparing to studies that used a 0-4 point Likert scale^14,16^, we applied a normalization equation **Score_4-pt_ = (4 x Score_100-pt_) / 100.**

*Modified Falls Efficacy Scale (mFES)*

The mFES form is provided on page 29 of **Supplementary Material 1.** The score is the average of the answered items.

*Prosthetic Limb User Survey of Mobility (PLUS-M)*

The PLUS-M provides a T-score, which is a standardized score centered on 50 with a SD of 10, corresponding to the mean of the development sample. The PLUS-M developers additionally report means and quartiles of various sub-populations, including TT, TF, bilateral, dysvascular, trauma, male, female, and age groups. In our study, we used the PLUS-M™ 12-item short form v1.2 and T-score conversion table which are available on the PLUS-M™ website^17,18^.

*Patient-Reported Outcomes Measurement Information System (PROMIS)*

In our study, we assessed five specific scales, using the curated instruments from the REDCap shared library, as indicated in parentheses: Mobility (PROMIS Bank v2.0 – Mobility Computer Adaptive Test); Physical Function with Mobility Aid (PROMIS Item Bank v1.0 – Physical Function with Mobility Aid – Computer Adaptive Test); Depression (PROMIS Item Bank v1.0 – Emotional Distress – Depression Short Form 8a); Ability to Participate in Social Roles and Activities (SRA; PROMIS Item Bank v2.0 – Ability to Participate in Social Roles and Activities – Short Form 8a); Satisfaction with SRA (PROMIS Item Bank v2.0 – Satisfaction with Social Roles and Activities – Short Form 8a).

*Patient Health Questionnaire 9 (PHQ-9)*

The PHQ-9 asks how often the respondent experienced each of 9 depressive symptoms over the preceding 2 weeks. A final question, which was not considered in the current report, asks how difficult any of the symptoms made it to do daily tasks. PHQ-9 scores of 0-4, 5-9, 10-14, 15-19, and 20-27 represent none/minimal, mild, moderate, moderately severe, and severe depression, respectively^19^. The PHQ-9 form is included in **Supplementary Material 1** (p. 30).

*Community Participation Indicators (CPI)*

The CPI is a self-reported measure that uses a scale of 0-7 days per week to assess how often a person participates in community activities^20,21^. It also includes a 48-item questionnaire that measures participation in activities deemed to be important, as well as the sense of control over their participation. The first part presents 20 community and household activities and for each asks 1) how often the respondent does the activity, 2) whether it is important, and 3) if they are doing it often enough^13,14^. The score of the first part is the percentage of important activities done enough (or too much), i.e.:

$$Score= \frac{number of important items done enough or too much}{number of important items} x 100$$

The second part gauges the respondent’s sense of involvement in life situations (14 items) and their control over participation (13 items). The form for both parts and the scoring guide of the second part are provided on pages 31-38 of **Supplementary Material 1.**

**References**

1. Gailey RS, Roach KE, Applegate EB, et al. The Amputee Mobility Predictor: An instrument to assess determinants of the lower-limb amputee’s ability to ambulate. *Arch Phys Med Rehabil*. 2002;83(5):613-627. doi:10.1053/apmr.2002.32309

2. Gailey RS, Scoville C, Gaunaurd IA, et al. Construct validity of Comprehensive High-Level Activity Mobility Predictor (CHAMP) for male servicemembers with traumatic lowerlimb loss. *J Rehabil Res Dev*. 2013;50(7):919-930. doi:10.1682/JRRD.2012.05.0100

3. Gaunaurd IA. *The Comprehensive High-Level Activity Mobility Predictor (CHAMP): A Performance-Based Assessment Instrument to Quantify High-Level Mobility in Service Members with Traumatic Lower Limb Loss*. http://scholarlyrepository.miami.edu/oa_dissertations

4. Gailey RS, Gaunaurd IA, Raya MA, et al. Development and reliability testing of the Comprehensive High-Level Activity Mobility Predictor (CHAMP) in male servicemembers with traumatic lower-limb loss. *J Rehabil Res Dev*. 2013;50(7):905-918. doi:10.1682/JRRD.2012.05.0099

5. Anton A, Legault Z, Dudek N. Validity of the Comprehensive High-Level Activity Mobility Predictor in a heterogeneous population with lower extremity amputations. *Prosthet Orthot Int*. 2020;44(2):60-65. doi:10.1177/0309364619887559

6. Gailey RS, Gaunaurd I, Morgan SJ, et al. A Comparison of the Two-Minute Walk Test (2MWT) and Comprehensive High-level Activity Mobility Predictor (CHAMP) in People with a Leg Prosthesis. *Clin Rehabil*. 2022;36(5):703-712. doi:10.1177/02692155211069323

7. Heinemann AW, Bode RK, O’reilly C. Development and measurement properties of the Orthotics and Prosthetics Users’ Survey (OPUS): a comprehensive set of clinical outcome instruments. *Prosthet Orthot Int*. 2003;27:191-206. doi:10.1080/03093640308726682

8. Heinemann AW, Fisher WP, Gershon R. Improving Health Care Quality With Outcomes Management. *Journal of Prosthetics and Orthotics*. 2006;18:46-50. doi:10.1097/00008526-200601001-00005

9. Heinemann AW, Gershon R, Fisher WP. Development and Application of the Orthotics and Prosthetics User Survey: Applications and Opportunities for Health Care Quality Improvement. *Journal of Prosthetics and Orthotics*. Published online 2006:80-85. doi:10.1097/00008526-200601001-00009

10. OrthoCare Innovations. Prosthesis Evaluation Questionnaire (PEQ). Accessed January 6, 2024. https://orthocareinnovations.com/wp-content/uploads/2021/11/PEQ.pdf

11. Miller WC, Deathe AB, Speechley M. Lower extremity prosthetic mobility: A comparison of 3 self-report scales. *Arch Phys Med Rehabil*. 2001;82(10):1432-1440. doi:10.1053/apmr.2001.25987

12. Legro MW, Reiber GD, Smith DG, De1 Aguila M, Larsen J, Boone D. Prosthesis Evaluation Questionnaire for Persons With Lower Limb Amputations: Assessing Prosthesis-Related Quality of Life. *Archives Physical Medicine and Rehabilitation* . 1998;79.

13. Boone DA, Coleman KL. Use of the Prosthesis Evaluation Questionnaire. *American Academy of Orthotists and Prosthetists*. Published online 2006:68-79.

14. Franchignoni F, Giordano A, Ferriero G, Orlandini D, Amoresano A, Perucca L. Measuring mobility in people with lower limb amputation: Rasch analysis of the mobility section of the prosthesis evaluation questionnaire. *J Rehabil Med*. 2007;39(2):138-144. doi:10.2340/16501977-0033

15. Resnik L, Borgia M. *Reliability of Outcome Measures for People With Lower-Limb Amputations: Distinguishing True Change From Statistical Error*.; 2011. https://academic.oup.com/ptj/article/91/4/555/2735043

16. Hafner BJ, Gaunaurd IA, Morgan SJ, Amtmann D, Salem R, Gailey RS. Construct Validity of the Prosthetic Limb Users Survey of Mobility (PLUS-M) in Adults With Lower Limb Amputation. In: *Archives of Physical Medicine and Rehabilitation*. Vol 98. W.B. Saunders; 2017:277-285. doi:10.1016/j.apmr.2016.07.026

17. PLUS-M^TM^. PLUS-M^TM^ User’s Guide, US English, Version 1.2. Accessed December 29, 2024. https://plus-m.org/files/PLUS-M_Users_Guide_US_ENGLISH_v1.2.pdf

18. PLUS-M^TM^. Prosthetic Limb Users Survey of Mobility (PLUS-M^TM^) 12-item Short Form (v1.2). Accessed December 29, 2020. https://plus-m.org/files/PLUS-M_12-Item_SF_US_ENGLISH_v1.2.pdf

19. Kroenke K, Spitzer RL, Williams JBW. The PHQ-9 Validity of a Brief Depression Severity Measure. *J Gen Intern Med*. 2001;16:606-613.

20. Heinemann AW, Lai JS, Magasi S, et al. Measuring participation enfranchisement. *Arch Phys Med Rehabil*. 2011;92(4):564-571. doi:10.1016/j.apmr.2010.07.220

21. Heinemann AW, Magasi S, Bode RK, et al. Measuring enfranchisement: Importance of and control over participation by people with disabilities. *Arch Phys Med Rehabil*. 2013;94(11):2157-2165. doi:10.1016/j.apmr.2013.05.017
