# Supplementary material for: References values and standardized testing protocols for performance-based and patient-reported outcome measures among individuals with lower limb amputation
Source: Front Rehabil Sci. 2026 Mar 13;7:1786298. doi: 10.3389/fresc.2026.1786298 (PMC13021846; doi:10.3389/fresc.2026.1786298)
Supplement: Supplementary file 3 [file Datasheet1.pdf]

Four Square Step Test (FSST)

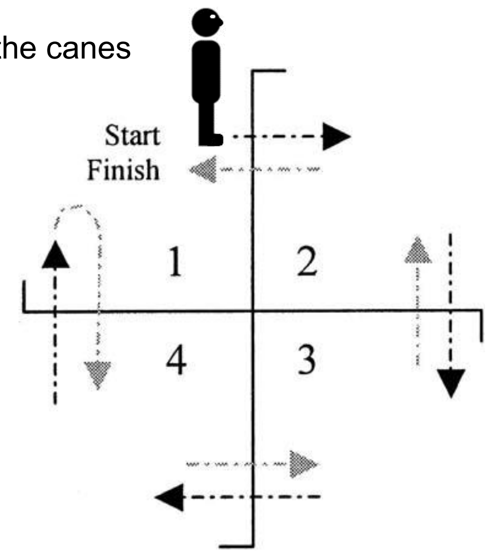

Instructions (derived from Dite and Temple 2002):

- Setup four canes like a cross on the floor with tips of the canes together. The direction and type of handle used is not important.

- Sequence: The subject starts in square 1, facing square 2. The subject steps forward into square 2, sideway to square 3, backward to square 4, sideway to square 1, sideway to square 4, forward to square 3, sideway to square 2, and backward to square 1.

- Standard instruction: "Try to complete the sequence as fast as possible without touching the sticks. Both feet must make contact with the floor in each square. If possible, face forward during the entire sequence."

- Demonstrate the sequence to the patient.

- Ask the patient to complete one practice trial to ensure the patient knows the sequence. Repeat the trial if the patient is unsuccessful at completing the sequence, loses balance, or contacts a cane during the trial.

Scoring:

- Two FSST are completed with the best time taken as the score.
- Stopwatch starts when the first foot contacts the floor in square 2. Stopwatch finishes when the last foot comes back to touch the floor in square 1.
- A score is still provided if the patient is unable to face forward during the entire sequence.

Assistive Device and/or Bracing Used:____

Trial 1: _______________sec. Trial 2: __________sec.

FSST Score (best timed trial): ____________sec.

Downloaded from www.rehabmeasures.org and revised

BERG BALANCE SCALE

*****THE STANDARDIZATION CONSIDERATIONS REFLECT REHABILITATION INSTITUTE OF CHICAGO STANDARDIZATION INSTRUCTIONS.**

To differentiate between items that indicate a patient can ***perform an activity with supervision vs perform it safely***, please think about the following definitions:

- Performs with supervision: The patient needs you nearby for any reason, including for safety (loss of balance, poor insight, poor cognition, etc). If you don't feel it is appropriate to walk away from the patient during the test item, please indicate the patient requires supervision
- Performs safely: the patient can perform the activity without assistance and without supervision. Only select that a patient can perform safely if you would feel comfortable walking away from the patient while he/she performs the activity that is being tested.

ITEM	Eval
1. **SITTING TO STANDING -** INSTRUCT: Please stand up. Try not to use your hands for support.<br>4) able to stand without using hands and stabilize independently<br>3) able to stand independently using hands<br>2) able to stand using hands after several tries<br>1) needs minimal aid to stand or to stabilize<br>0) needs moderate or maximal assist to stand<br><br>****Standardization considerations: If a patient is uses a wheelchair, it is OK to use the wheelchair for this item. If a patient does not use a wheelchair, use a standard testing chair with arms in your clinic.*	4<br>3<br>2<br>1<br>0
2. **STANDING UNSUPPORTED -** INSTRUCT: Please stand for two min without holding on.<br>4) able to stand safely 2 minutes<br>3) able to stand 2 minutes with supervision<br>2) able to stand 30 seconds unsupported<br>1) needs several tries to stand 30 seconds unsupported<br>0) unable to stand 30 seconds unassisted<br>***If a subject is able to stand 2 min unsupported, score full points for #3 and proceed to #4.***<br><br>*Standardization considerations:*<br>**** Do not provide explicit instructions on how to place feet to obtain balance. It is OK to state "Get your balance," however, does not instruct the patient on how to do this.*<br>****Unsupported (as described in score 2 & 3) means that the PT should NOT have any hands on the patient (even if only providing contact guard assistance).*	4<br>3<br>2<br>1<br>0
3. **SITTING WITH BACK UNSUPPORTED BUT FEET SUPPORTED ON FLOOR OR ON A STOOL -** INSTRUCTIONS: Please sit with arms folded for 2 minutes.<br>4) able to sit safely and securely 2 minutes<br>3) able to sit 2 minutes under supervision<br>2) able to sit 30 seconds<br>1) able to sit 10 seconds<br>0) unable to sit without support 10 seconds<br><br>***Standardization considerations: The thigh of the legs should be completely supported (as opposed to scooted to the edge with only ischial tuborosities supported). Back should NOT be supported, feet should be on the floor.	4<br>3<br>2<br>1<br>0

4. **STANDING TO SITTING -** INSTRUCTIONS: Please sit down.<br>4) sits safely with minimal use of hands<br>3) controls descent by using hands<br>2) uses back of legs against chair to control descent<br>1) sits independently but has uncontrolled descent<br>0) needs assistance to sit<br><br>****Standardization considerations: To score a 4 on this item, the patient can ONLY use hands for guidance into the chair. If any weight is transferred onto the arm, then the patient can score of 3 on the item at best. If there is ANY increase in velocity while the patient is sitting, consider it an "uncontrolled descent"*	 4<br>3<br>2<br>1<br>0
5. **TRANSFERS -** INSTRUCTIONS: Arrange chairs(s) for a pivot transfer. Ask subject to transfer one way toward a seat with armrests and one way toward a seat without armrests. You may use two chairs (one with and one without armrests) or a bed and a chair.<br>4) able to transfer safely with minor use of hands<br>3) able to transfer safely definite need of hands<br>2) able to transfer with verbal cueing and/or supervision<br>1) needs one person to assist<br>0) needs two people to assist or supervise to be safe<br><br>****Standardization considerations: If a patient is uses a wheelchair, it is OK to use the wheelchair and a mat or another standard chair for this item. If a patient does not use a wheelchair, use a standard testing chair with arms and a mat or a another chair in your clinic.*	 <br><br>4<br>3<br>2<br>1<br>0
6. **STANDING UNSUPPORTED WITH EYES CLOSED**<br>INSTRUCTIONS: Please close your eyes and stand still for 10 seconds.<br>4) able to stand 10 seconds safely<br>3) able to stand 10 seconds with supervision<br>2) able to stand 3 seconds<br>1) unable to keep eyes closed 3 seconds but stays steady<br>0) needs help to keep from falling<br><br>*Standardization considerations:*<br>**** Do not provide explicit instructions on how to place feet to obtain balance. It is OK to state "Get your balance," however, does not instruct the patient on how to do this.*	 <br>4<br>3<br>2<br>1<br>0
7. **STANDING UNSUPPORTED WITH FEET TOGETHER**<br>INSTRUCTIONS: Place your feet together and stand without holding.<br>4) able to place feet together independently and stand 1 minute safely<br>3) able to place feet together Independently and stand for 1 min with supervision<br>2) able to place feet together independently and to hold for 30 seconds<br>1) needs help to attain position but able to stand 15 seconds feet together<br>0) needs help to attain position and unable to hold for 15 seconds<br><br>****Standardization considerations: To score a 1 on this item, the patient can use assistance from an assistive device or the examiner, however, the patient must then let go and hold for 15 seconds in the position.*	 <br>4<br>3<br>2<br>1<br>0

8. **REACHING FORWARD WITH OUTSTRETCHED ARM WHILE STANDING**<br>INSTRUCTIONS: Lift arm to 90 degrees. Stretch out your fingers and reach forward as far as you can. (Examiner places a ruler at end of fingertips when arm is at 90 degrees. Fingers should not touch the ruler while reaching forward. The recorded measure is the distance forward that the finger reach while the subject is in the most forward lean position. When possible, ask subject to use both arms when reaching to avoid rotation of the trunk.)<br>4) can reach forward confidently >25 cm (10 inches)<br>3) can reach forward >12.5 cm safely (5 inches)<br>2) can reach forward >5 cm safely (2 inches)<br>1) reaches forward but needs supervision<br>0) loses balance while trying/ requires external support<br><br>****Standardization considerations: Shoulders must remain even during the forward lean, no twisting of the trunk is allowed. The strong UE should be used for reaching, and the other should remain at the side.*	4<br>3<br>2<br>1<br>0
9. **PICK UP OBJECT FROM THE FLOOR FROM A STANDING POSITION**<br>INSTRUCTIONS: Pick up the shoe/slipper which is placed in front of your feet.<br>4) able to pick up slipper safely and easily<br>3) able to pick up slipper but needs supervision<br>2) unable to pick up but reaches 1-2 in. from slipper, keeps balance independently<br>1) unable to pick up and needs supervision while trying<br>0) unable to try/needs assist to keep from losing balance or falling<br><br>****Standardization considerations: Use an empty tissue box for testing instead of the shoe/slipper.*	4<br>3<br>2<br>1<br>0
10. **TURNING TO LOOK BEHIND OVER LEFT AND RIGHT SHOULDERS WHILE STANDING -** INSTRUCTIONS: Turn to look **directly** behind you over toward left shoulder. Repeat to the right. Examiner may pick an object to look at directly behind the subject to encourage a better twist turn.<br>4) looks behind from both sides and weight shifts well<br>3) looks behind one side only other side shows less weight shift<br>2) turns sideways only but maintains balance<br>1) needs supervision when turning<br>0) needs assist to keep from losing balance or falling<br><br>*Standardization considerations:*<br>**** Do not provide explicit instructions on how to place feet to obtain balance. It is OK to state "Get your balance," however, does not instruct the patient on how to do this.*<br>****Select a target directly behind the patient to encourage a complete twist.*	4<br>3<br>2<br>1<br>0
11. **TURN 360 DEGREES -** INSTRUCTIONS: Turn completely around in a full circle. Pause. Then turn a full circle in the other direction.<br>4) able to turn 360 degrees safely in 4 seconds or less<br>3) able to turn 360 degrees safely one side only in 4 seconds or less<br>2) able to turn 360 degrees safely but slowly<br>1) needs close supervision or verbal cueing<br>0) needs assistance while turning<br><br>*Standardization considerations:*<br>****No UE support is allowed during turning*<br>****To score a 4 on the item, the patient must turn safely in 4 seconds PER side.*	4<br>3<br>2<br>1<br>0

12. **PLACING ALTERNATE FOOT ON STEP OR STOOL WHILE STANDING UNSUPPORTED - I**NSTRUCTIONS: Place each foot alternately on the step/stool (6 inches). Continue until each foot has touched the step/stool four times.<br>4) able to stand independently and safely and complete 8 steps in 20 seconds<br>3) able to stand independently and complete 8 steps >20 seconds<br>2) able to complete 4 steps without aid with supervision<br>1) able to complete >2 steps needs minimal assist<br>0) needs assistance to keep from falling/unable to try<br><br>*Standardization considerations:*<br>****A 6 inch stool should be used*<br>****The patient should actually tap foot on the stool, there should be NO weight transfer onto foot on the box.*	 <br><br>4<br>3<br>2<br>1<br>0
13. **STANDING UNSUPPORTED ONE FOOT IN FRONT -** INSTRUCTIONS: (DEMONSTRATE TO SUBJECT) Place one foot directly in front of the other. If you feel that you cannot place your foot directly in front, try to step far enough ahead that the heel of your forward foot is ahead of the toes of the other foot. (To score 3 points, the length of the step should exceed the length of the other foot and the width of the stance should approximate the subject's normal stride width)<br>4) able to place foot tandem independently and hold 30 seconds<br>3) able to place foot ahead of other independently and hold 30 seconds<br>2) able to take small step independently and hold 30 seconds<br>1) needs help to step but can hold 15 seconds<br>0) loses balance while stepping or standing<br><br>*Standardization considerations:*<br>****The weaker leg should be supporting the weight (strong leg steps in front)* *For our study consider the prosthesis side the weaker leg.*<br>****3 tries are allowed on this item. If a patient attempts tandem, but is unable to hold for 30 seconds, re-instruct for foot-ahead (score 3). If unsuccessful, re-instruct for small step (score 2).*<br>****To score a 1 on this item, the patient can use assistance from an assistive device or the*	 <br><br><br><br>4<br>3<br>2<br>1<br>0
14. **STANDING ON ONE LEG -** INSTRUCT: Stand on one leg as long as you can without holding.<br>4) able to lift leg independently and hold >10 seconds<br>3) able to lift leg independently and hold 5-10 seconds<br>2) able to lift leg independently and hold = or >3 seconds<br>1) tries to lift leg unable to hold 3 seconds but remains standing independently<br>0) unable to try or needs assist to prevent fall<br><br>*Standardization considerations:*<br>****The weaker leg should be supporting the weight (strong leg steps in front)* *For our study consider the prosthesis side the weaker leg.*	 4<br>3<br>2<br>1<br>0
**TOTAL SCORE:**	

Functional Gait Assessment

*****THE STANDARDIZATION CONSIDERATIONS REFLECT REHABILITATION INSTITUTE OF CHICAGO STANDARDIZATION INSTRUCTIONS.**

ITEM	Eval
**1. GAIT LEVEL SURFACE -** Instructions: Walk at your normal speed from here to the next mark (6 m [20 ft]).<br><br>Grading: Mark the highest category that applies.<br><br>3) Normal: Walks 6 m (20 ft) in less than 5.5 seconds, no assistive devices, good speed, no evidence for imbalance, normal gait pattern, deviates no more than 15.24 cm (6 in) outside of the 30.48-cm (12-in) walkway width.<br><br>2) Mild impairment: Walks 6 m (20 ft) in less than 7 seconds but greater than 5.5 seconds, uses assistive device, slower speed, mild gait deviations, or deviates 15.24–25.4 cm (6–10 in) outside of the 30.48-cm (12-in) walkway width.<br><br>1) Moderate impairment—Walks 6 m (20 ft), slow speed, abnormal gait pattern, evidence for imbalance, or deviates 25.4– 38.1 cm (10–15 in) outside of the 30.48- cm (12-in) walkway width. Requires more than 7 seconds to ambulate 6 m (20 ft).<br><br>0) Severe impairment—Cannot walk 6 m (20 ft) without assistance, severe gait deviations or imbalance, deviates greater than 38.1 cm (15 in) outside of the 30.48- cm (12-in) walkway width or reaches and touches the wall.<br><br>****Standardization considerations: Use a straight path, no curves.*<br>****If an assistive device is used, the highest score that patient can receive is a 2.*	3<br>2<br>1<br>0
**2. CHANGE IN GAIT SPEED -** Instructions: Begin walking at your normal pace (for 1.5 m [5 ft]). When I tell you "go," walk as fast as you can (for 1.5 m [5 ft]). When I tell you "slow," walk as slowly as you can (for 1.5 m [5 ft]).<br><br>Grading: Mark the highest category that applies.<br><br>3) Normal—Able to smoothly change walking speed without loss of balance or gait deviation. Shows a significant difference in walking speeds between normal, fast, and slow speeds. Deviates no more than 15.24 cm (6 in) outside of the 30.48-cm (12-in) walkway width.<br><br>2) Mild impairment—Is able to change speed but demonstrates mild gait deviations, deviates 15.24–25.4 cm (6–10 in) outside of the 30.48-cm (12-in) walkway width, or no gait deviations but unable to achieve a significant change in velocity, or uses an assistive device.<br><br>1) Moderate impairment—Makes only minor adjustments to walking speed, or accomplishes a change in speed with significant gait deviations, deviates 25.4–38.1 cm (10–15 in) outside the 30.48-cm (12-in) walkway width, or changes speed but loses balance but is able to recover and continue walking.<br><br>0) Severe impairment—Cannot change speeds, deviates greater than 38.1 cm (15 in) outside 30.48-cm (12-in) walkway width, or loses balance and has to reach for wall or be caught.<br><br>****Standardization considerations: It is OK to demonstrate and provide simpler instructions.*<br>****If an assistive device is used, the highest score that patient can receive is a 2.*	3<br>2<br>1<br>0

**3. GAIT WITH HORIZONTAL HEAD TURNS.** Instructions: Walk from here to the next mark 6 m (20 ft) away. Begin walking at your normal pace. Keep walking straight; after 3 steps, turn your head to the right and keep walking straight while looking to the right. After 3 more steps, turn your head to the left and keep walking straight while looking left. Continue alternating looking right and left every 3 steps until you have completed 2 repetitions in each direction.<br><br>Grading: Mark the highest category that applies.<br>3) Normal—Performs head turns smoothly with no change in gait. Deviates no more than 15.24 cm (6 in) outside 30.48-cm (12-in) walkway width.<br><br>2) Mild impairment—Performs head turns smoothly with slight change in gait velocity (eg, minor disruption to smooth gait path), deviates 15.24–25.4 cm (6–10 in) outside 30.48-cm (12-in) walkway width or uses assistive device.<br><br>1) Moderate impairment—Performs head turns with moderate change in gait velocity, slows down, deviates 25.4–38.1 cm (10–15 in) outside 30.48-cm (12-in) walkway width but recovers, can continue to walk.<br><br>0) Severe impairment—Performs task with severe disruption of gait(eg, staggers 38.1 cm [15 in] outside 30.48-cm (12-in) walkway width, loses balance, stops, or reaches for wall).<br><br>****Standardization considerations: It is OK to demonstrate and provide simpler instructions, and provide cues while walking.*<br>****If an assistive device is used, the highest score that patient can receive is a 2.*<br>**** If patient has cervical collar, it is OK to rotate at trunk. If patient has full spinal precautions, score this item as 0.*	3<br>2<br>1<br>0
4. **GAIT WITH VERTICAL HEAD TURNS.** Instructions: Walk from here to the next mark (6 m [20 ft]). Begin walking at your normal pace. Keep walking straight; after 3 steps, tip your head up and keep walking straight while looking up. After 3 more steps, tip your head down, keep walking straight while looking down. Continue alternating looking up and down every 3 steps until you have completed 2 repetitions in each direction.<br><br>Grading: Mark the highest category that applies.<br><br>3) Normal—Performs head turns with no change in gait. Deviates no more than 15.24 cm (6 in) outside 30.48-cm (12-in) walkway width.<br><br>2) Mild impairment—Performs task with slight change in gait velocity (eg, minor disruption to smooth gait path), deviates 15.24–25.4 cm (6–10 in) outside 30.48-cm (12-in) walkway width or uses assistive device.<br><br>1) Moderate impairment—Performs task with moderate change in gait velocity, slows down, deviates 25.4–38.1 cm (10–15 in) outside 30.48-cm (12-in) walkway width but recovers, can continue to walk.<br><br>0) Severe impairment—Performs task with severe disruption of gait (eg, staggers 38.1 cm [15 in] outside 30.48-cm (12-in) walkway width, loses balance, stops, reaches for wall).<br><br>****Standardization considerations: It is OK to demonstrate and provide simpler instructions, and provide cues while walking.*<br>****If an assistive device is used, the highest score that patient can receive is a 2.*<br>**** If patient has cervical collar, it is OK to rotate at trunk. If patient has full spinal precautions, score this item as 0.*	3<br>2<br>1<br>0

**5. GAIT AND PIVOT TURN -** Instructions: Begin with walking at your normal pace. When I tell you, "turn and stop," turn as quickly as you can to face the opposite direction and stop. Grading: Mark the highest category that applies.<br><br>3) Normal—Pivot turns safely within 3 seconds and stops quickly with no loss of balance.<br><br>2) Mild impairment—Pivot turns safely in >3 seconds and stops with no loss of balance, or pivot turns safely within 3 seconds and stops with mild imbalance, requires small steps to catch balance.<br><br>1) Moderate impairment—Turns slowly, requires verbal cueing, or requires several small steps to catch balance following turn and stop.<br><br>0) Severe impairment—Cannot turn safely, requires assistance to turn and stop.<br><br>****Standardization considerations: It is OK to demonstrate item, patient can take multiple steps to make the turn (doesn't have to be a pivot turn). Patient can walk as far as needed to get to normal pace prior to saying "turn and stop."*<br>****If the patient is unable to complete without an AD, score a 0 on this item.*	3<br>2<br>1<br>0
**6. STEP OVER OBSTACLE -** Instructions: Begin walking at your normal speed. When you come to the shoe box, step over it, not around it, and keep walking.<br><br>Grading: Mark the highest category that applies.<br><br>3) Normal—Is able to step over 2 stacked shoe boxes taped together (22.86 cm [9 in] total height) without changing gait speed; no evidence of imbalance.<br><br>2) Mild impairment—Is able to step over one shoe box (11.43 cm [4.5 in] total height) without changing gait speed; no evidence of imbalance.<br><br>1) Moderate impairment—Is able to step over one shoe box (11.43 cm [4.5 in] total height) but must slow down and adjust steps to clear box safely. May require verbal cueing.<br><br>0) Severe impairment—Cannot perform without assistance.<br><br>****Standardization considerations: It is OK to demonstrate item. Any item that is the length of a shoebox, reasonable width, and 9 inches tall can be used.*<br>****If the patient is unable to complete without an AD, score a 0 on this item.*	3<br>2<br>1<br>0
**7. GAIT WITH NARROW BASE OF SUPPORT -** Instructions: Walk on the floor with arms folded across the chest, feet aligned heel to toe in tandem for a distance of 3.6 m [12 ft]. The number of steps taken in a straight line are counted for a maximum of 10 steps. Grading: Mark the highest category that applies.<br><br>3) Normal—Is able to ambulate for 10 steps heel to toe with no staggering.<br><br>2) Mild impairment—Ambulates 7–9 steps.<br><br>1) Moderate impairment—Ambulates 4–7 steps.<br><br>0) Severe impairment—Ambulates less than 4 steps heel to toe or cannot perform without assistance.<br><br>****Standardization considerations: Impaired UE can be at the patient's side.*<br>****If the patient is unable to complete without an AD, score a 0 on this item.*<br>****Steps must be consecutive to count.*	3<br>2<br>1<br>0

**8. GAIT WITH EYES CLOSED -** Instructions: Walk at your normal speed from here to the next mark (6 m [20 ft]) with your eyes closed. Grading: Mark the highest category that applies.<br>3) Normal—Walks 6 m (20 ft), no assistive devices, good speed, no evidence of imbalance, normal gait pattern, deviates no more than 15.24 cm (6 in) outside 30.48-cm (12-in) walkway width. Ambulates 6 m (20 ft) in less than 7 seconds.<br>2) Mild impairment—Walks 6 m (20 ft), uses assistive device, slower speed, mild gait deviations, deviates 15.24–25.4 cm (6–10 in) outside 30.48-cm (12-in) walkway width. Ambulates 6 m (20 ft) in less than 9 seconds but greater than 7 seconds.<br>1) Moderate impairment—Walks 6 m (20 ft), slow speed, abnormal gait pattern, evidence for imbalance, deviates 25.4–38.1cm (10–15 in) outside 30.48-cm (12-in) walkway width. Requires more than 9 seconds to ambulate 6 m (20 ft).<br>0) Severe impairment—Cannot walk 6 m (20 ft) without assistance, severe gait deviations or imbalance, deviates greater than 38.1 cm (15 in) outside 30.48-cm (12-in) walkway width or will not attempt task.<br>****Standardization considerations: It is OK to demonstrate to the patient.*<br>****If an assistive device is used, the highest score that patient can receive is a 2.*	3<br>2<br>1<br>0
**9. AMBULATING BACKWARDS -** Instructions: Walk backwards until I tell you to stop. Grading: Mark the highest category that applies.<br>3) Normal—Walks 6 m (20 ft), no assistive devices, good speed, no evidence for imbalance, normal gait pattern, deviates no more than 15.24 cm (6 in) outside 30.48-cm (12-in) walkway width.<br>2) Mild impairment—Walks 6 m (20 ft), uses assistive device, slower speed, mild gait deviations, deviates 15.24–25.4 cm (6–10 in) outside 30.48-cm (12-in) walkway width.<br>1) Moderate impairment—Walks 6 m (20 ft), slow speed, abnormal gait pattern, evidence for imbalance, deviates 25.4–38.1 cm (10–15 in) outside 30.48-cm (12-in) walkway width.<br>0) Severe impairment—Cannot walk 6 m (20 ft) without assistance, severe gait deviations or imbalance, deviates greater than 38.1cm (15 in) outside 30.48-cm (12-in) walkway width or will not attempt task.<br>****Standardization considerations: It is OK to demonstrate to the patient.*<br>****If an assistive device is used, the highest score that patient can receive is a 2.*	3<br>2<br>1<br>0
**10. STEPS -** Instructions: Walk up these stairs as you would at home (ie, using the rail if necessary). At the top turn around and walk down. Grading: Mark the highest category that applies.<br>3) Normal—Alternating feet, no rail.<br>2) Mild impairment—Alternating feet, must use rail.<br>1) Moderate impairment—Two feet to a stair; must use rail.<br>0) Severe impairment—Cannot do safely.<br>****Standardization considerations: Steps need to have bilateral rails.*<br>****If the patient is unable to complete without an AD, score a 0 on this item.*<br>****Any number of steps greater than 4 can be utilized.*<br>****Keep number of stairs consistent from test to test.*	3<br>2<br>1<br>0
**Total Score:**	

AMPUTEE MOBILITY PREDICTOR ASSESSMENT TOOL

Initial instructions: Client is seated in a hard chair with arms. The following manoeuvres are tested with or without the use of the prosthesis. Advise the person of each task or group of tasks prior to performance. Please avoid unnecessary chatter throughout the test. Safety First, no task should be performed if either the tester or client is uncertain of a safe outcome.

The **Right Limb** is: ❒ PF ❒ TT ❒ KD ❒ TF ❒ HD ❒ intact

The **Left Limb** is: ❒ PF ❒ TT ❒ KD ❒ TF ❒ HD ❒ intact

Abbreviation: PF = partial foot; TT = transtibial; KD = knee disarticulation; TF = transfemoral; HD = hip disarticulation

**1.** Sitting Balance:<br>Sit forward in a chair with arms folded across chest for 60s.	Cannot sit upright independently for 60s<br>Can sit upright independently for 60s	= 0<br>= 1	_______
**2.** Sitting reach:<br>Reach forwards and grasp the ruler. (Tester holds ruler 12in beyond extended arms midline to the sternum)	Does not attempt<br>Cannot grasp or requires arm support<br>Reaches forward and successfully grasps item.	= 0<br>= 1<br>= 2	_______
**3.** Chair to chair transfer:<br>2 chairs at 90°. Pt. may choose direction and use their upper limbs.	Cannot do or requires physical assistance<br>Performs independently, but appears unsteady<br>Performs independently, appears to be steady and safe	= 0<br>= 1<br>= 2	_______
**4.** Arises from a chair:<br>Ask pt. to fold arms across chest and stand. If unable, use arms or assistive device.	Unable without help (physical assistance)<br>Able, uses arms/assist device to help<br>Able, without using arms	= 0<br>= 1<br>= 2	_______
**5.** Attempts to arise from a chair:<br>(stopwatch ready) If attempt in no. 4. was without arms then ignore and allow another attempt without penalty.	Unable without help (physical assistance)<br>Able requires >1 attempt<br>Able to rise one attempt	= 0<br>= 1<br>= 2	_______
**6.** Immediate Standing Balance:<br>(**first 5s**) Begin timing immediately.	Unsteady (staggers, moves foot, sways)<br>Steady using walking aid or other support<br>Steady without walker or other support	= 0<br>= 1<br>= 2	_______
**7.** Standing Balance (30s):<br>(stopwatch ready) For item no.'s 7 & 8, first attempt is without assistive device. If support is required allow after first attempt	Unsteady<br>Steady but uses walking aid or other support<br>Standing without support	= 0<br>= 1<br>= 2	_______
**8.** Single limb standing balance:<br>(stopwatch ready) Time the duration of single limb standing on both the sound and prosthetic limb up to 30s.<br><br>Grade the quality, not the time.<br><br>**Eliminate item 8 for AMPnoPRO**<br><br>Sound side ______ seconds<br><br>Prosthetic side ______ seconds	**Non-prosthetic side**<br>Unsteady<br>Steady but uses walking aid or other support for 30s<br>Single-limb standing without support for 30s<br><br>**Prosthetic Side**<br>Unsteady<br>Steady but uses walking aid or other support for 30s<br>Single-limb standing without support for 30s	= 0<br>= 1<br>= 2<br><br><br>= 0<br>= 1<br>= 2	_______<br><br><br><br><br>_______
**9.** Standing reach:<br>Reach forward and grasp the ruler. (Tester holds ruler 12in beyond extended arm(s) midline to the sternum)	Does not attempt<br>Cannot grasp or requires arm support on assistive device<br>Reaches forward and successfully grasps item no support	= 0<br>= 1<br>= 2	______
**10.** Nudge test:<br>With feet as close together as possible, examiner pushes lightly on pt.'s sternum with palm of hand 3 times (toes should rise)	Begins to fall<br>Staggers, grabs, catches self ore uses assistive device<br>Steady	= 0<br>= 1<br>= 2	_______
**11.** Eyes Closed:<br>(at maximum position #7) If support is required grade as unsteady.	Unsteady or grips assistive device<br>Steady without any use of assistive device	= 0<br>= 1	_______

Date: ______________ Tester Initials: ___________ Subject ID: _________

**12.** Pick up objects off the floor:<br>Pick up a pencil off the floor placed midline 12in in front of foot.	Unable to pick up object and return to standing<br>Performs with some help (table, chair, walking aid etc)<br>Performs independently (without help)	= 0<br>= 1<br>= 2	_______
**13.** Sitting down:<br>Ask pt. to fold arms across chest and sit. If unable, use arm or assistive device.	Unsafe (misjudged distance, falls into chair)<br>Uses arms, assistive device or not a smooth motion<br>Safe, smooth motion	= 0<br>= 1<br>= 2	_______
**14.** Initiation of gait:<br>(immediately after told to "go")	Any hesitancy or multiple attempts to start<br>No hesitancy	= 0<br>= 1	_______
**15.** Step length and height:<br>Walk a measured distance of 12ft twice (up and back). Four scores are required or two scores (a. & b.) for each leg. "Marked deviation" is defined as extreme substitute movements to avoid clearing the floor.	**a. Swing Foot**<br>Does not advance a minimum of 12in = 0<br>Advances a minimum of 12in = 1<br><br>**b. Foot Clearance**<br>Foot does not completely clear floor without deviation = 0<br>Foot completely clears floor without marked deviation = 1	Prosthesis<br>_______<br><br>_______	Sound<br>______<br><br>______
**16.** Step Continuity	Stopping or discontinuity between steps (stop & go gait)<br>Steps appear continuous	= 0<br>= 1	_______
**17.** Turning:<br>180 degree turn when returning to chair.	Unable to turn, requires intervention to prevent falling<br>Greater than three steps but completes task without intervention<br>No more than three continuous steps with or without assistive aid	= 0<br>= 1<br>= 2	_______
**18.** Variable cadence:<br>Walk a distance of 12ft fast as possible safely 4 times. (Speeds may vary from slow to fast and fast to slow varying cadence)	Unable to vary cadence in a controlled manner<br>Asymmetrical increase in cadence controlled manner<br>Symmetrical increase in speed in a controlled manner	= 0<br>= 1<br>= 2	_______
**19.** Stepping over an obstacle:<br>Place a movable box of 4in in height in the walking path.	Cannot step over the box<br>Catches foot, interrupts stride<br>Steps over without interrupting stride	= 0<br>= 1<br>= 2	_______
**20.** Stairs (must have at least 2 steps):<br>Try to go up and down these stairs without holding on to the railing. Don't hesitate to permit pt. to hold on to rail. Safety First, if examiner feels that any risk in involved omit and score as 0.	**Ascending**<br>Unsteady, cannot do<br>One step at a time, or must hold on to railing or device<br>Step over step, does not hold onto the railing or device<br><br>**Descending**<br>Unsteady, cannot do<br>One step at a time, or must hold on to railing or device<br>Step over step, does not hold onto the railing or device	 = 0<br>= 1<br>= 2<br><br><br>= 0<br>= 1<br>= 2	_______<br><br>_______
**21.** Assistive device selection:<br>Add points for the use of an assistive device if used for two or more items. If testing without prosthesis use of appropriate assistive device is mandatory.	Bed bound<br>Wheelchair / Parallel Bars<br>Walker<br>Crutches (axillary or forearm)<br>Cane (straight or quad)<br>None	= 0<br>= 1<br>= 2<br>= 3<br>= 4<br>= 5	_______

Total Score AMPnoPRO _______/43

AMPPRO _______/47

Test: ☐ no prosthesis ☐ with prosthesis

K LEVEL (converted from AMP score)

AMPnoPRO ☐ K0 (0-8) ☐ K1 (9-20) ☐ K2 (21-28) ☐ K3 (29-36) ☐ K4 (37-43)

AMPPRO ☐ K1 (15-26) ☐ K2 (27-36) ☐ K3 (37-42) ☐ K4 (43-47)

Appendix G

The Comprehensive High-Level Activity Mobility Predictor (CHAMP) Instructions

**General Rules for Testers**	
1. Do not motivate or give suggestions to the participants.<br>2. The Tester will: 1) read the instructions word for word and 2) provide a walk through demonstration of each of the five test items.<br>3. Each participant is given at least 2 trials to complete each of the five test items. If they are unable to complete the test in two trials because of a disqualification, a third trial is offered.<br>4. If the participant falls during testing, a third trial is offered.<br>5. No more than 3 trials are to be offered for any of the test items.	
**1. Single Limb Stance (SLS):**	
**Verbal Instructions:**	
• On the command "Ready", fold arms across your chest<br>• When you are ready, lift your foot above the 15 cm cone or box<br>• If your foot falls below the 15 cm cone or box, you will be asked to raise it<br>• Time will be stopped if:<br>o Your foot touches the floor<br>o You do not maintain your foot above the 15 cm cone or box<br>o Arms come un-crossed<br>o The stationary foot loses contact with the floor (i.e., hopping)<br>o You achieve 30 seconds<br>• You will take a 30 second rest period after each trial	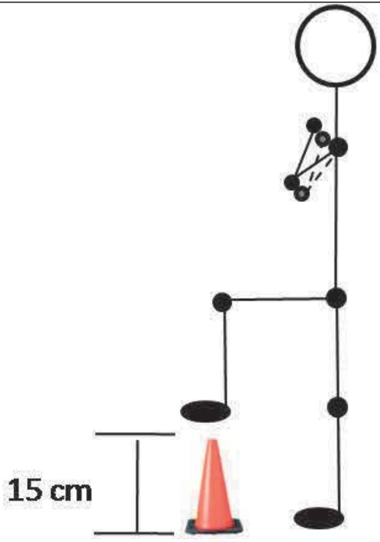

<table>
<tr><td colspan="2">3. Edgren Side Step Test:</td></tr>
<tr><td><u>Verbal Instructions:</u></td><td></td></tr>
<tr><td>
• The diagram illustrates the path you will complete for this test
• On the command “Ready” you will assume a standing position outside the far left cone
• On the command “Set”, prepare to sidestep
• On the command “Go”, you will sidestep to the right
• Sidestep to the right until your right foot has touched or crossed the right outside tape mark
• Then sidestep to the left until your left foot has touched or crossed the left outside tape mark
• Repeat this procedure as fast as possible in 10 seconds
• Points are awarded based on the number of cones you cross in 10 seconds
• If you fail to reach the outside cones, 1 point will not be awarded
• You will score a 0 and be asked to repeat the test, if:
    o You fail to keep your trunk and feet pointing forward at all times
    o You cross your legs
• You will take a 60 second rest period after each trial
</td><td>
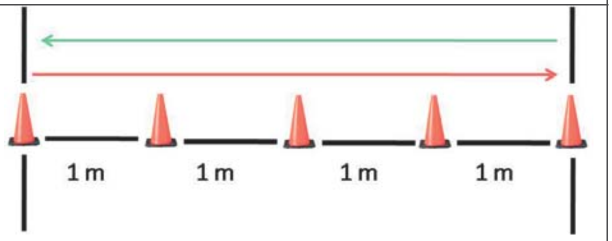

</td></tr>
</table>

4. T-Test:	
**Verbal Instructions:**	
• The diagram illustrates the path you will complete for this test<br>• On the command "Ready" you will assume a standing position behind the starting line<br>• On the command "Set", prepare to start<br>• On the command "Go", you will run or move as quickly as possible forward to the center cone (#1 cone)<br>• Sidestep right to the right cone (#2 cone)<br>• One foot must touch or cross the tape mark<br>• Sidestep left to the left cone (#3 cone)<br>• One foot must touch or cross the tape mark<br>• Sidestep right back to the center cone (#1 cone)<br>• One foot must touch or cross the tape mark<br>• Run or move as quickly as possible backwards to the finish line<br>• Your total time of completion of the T-Test will be recorded<br>• You will score a 0 and be asked to repeat the test, if:<br>o You fail to run the course as instructed<br>o You fail to reach the end lines<br>o You fail to complete the course<br>o You move any cones<br>o You fail to keep your trunk and feet pointing forward at all times or you cross your legs more than once<br>• You will take a 60 second rest period after each trial	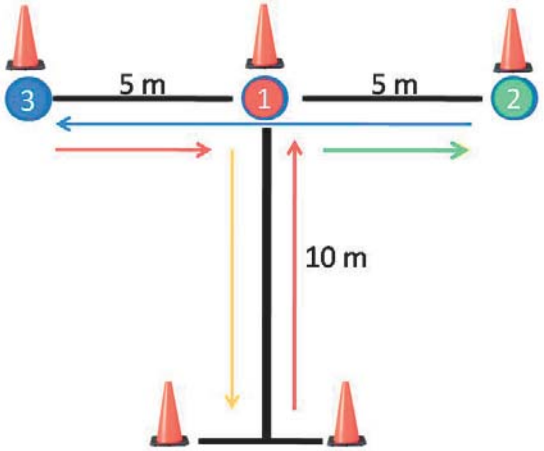

**<u>5. Illinois Agility Test</u>:**	
**Verbal Instructions:**	
• The diagram illustrates the path you will complete for this test<br>• On the command "Ready" lie on your stomach behind the starting line with your arms at your side and your head turned to the side or facing forward.<br>• On the command "Set", prepare to start, but you may NOT move your hands from your side<br>• On the command "Go", get up and run or move as quickly as possible to the first tape mark<br>• One foot must touch or cross the tape mark<br>• Turn around and run or move as quickly as possible back to the first center cone<br>• Weave up and back through the 4 center cones<br>• Run or move as quickly as possible to the second tape mark on the far line<br>• One foot must touch or cross the tape mark<br>• Turn around and run or move as quickly as possible across the finish cone<br>• Your total time for completion will be recorded<br>• You will score a 0, and be asked to repeat the test if:<br>○ You fail to run the course as instructed<br>○ You fail to reach the end lines<br>○ You fail to complete the course<br>○ You move any cone<br>• You will take a 60 second rest period after each trial	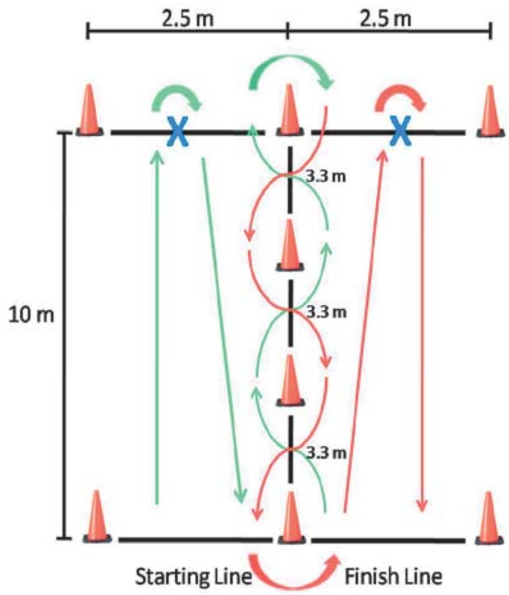

	SLS (s)	ESST (m)	T-Test (s)	IAT (s)
**Test Score**	**Time Score Range**	**Point Score Range**	**Time Score Range**	**Time Score Range**
	**Calculated based on a 3.3 second interval**	**Calculated based on a 3 meter interval**	**Calculated based on a 5.6 second interval**	**Calculated based on a 5.5 second interval**
**0**	0	< 5	>124	>65.4
**0.5**	0.1-3.3			
**1**	3.4-6.6	5 -7	50.7-123.9	60-65.4
**1.5**	6.7-10.0			
**2**	10.1-13.3	8 - 10	45.7-50.6	59.9-54.5
**2.5**	13.4-16.6			
**3**	16.7-19.9	11 -13	45.6-40.8	54.4-49.0
**3.5**	20-23.2			
**4**	23.3-26.5	14 -16	40.7-36.0	48.9-43.5
**4.5**	26.6-29.8			
**5**	29.9-33.1	17-19	35.9-31.1	43.4-38.0
**5.5**	33.2-36.4			
**6**	36.5-39.7	20-22	31.0-26.2	37.9-32.5
**6.5**	39.8-43			
**7**	43.1-46.3	23-25	26.1-21.3	32.4-27.0
**7.5**	46.4-49.6			
**8**	49.7-52.9	26-28	21.2-16.5	26.9-21.5
**8.5**	53.0-56.2			
**9**	56.3-59.5	29-31	16.4-11.6	21.4-15.9
**10**	60	≥32	<11.6	<15.9

Appendix J

The Comprehensive High-Level Activity Mobility Predictor (CHAMP)

			**Date:**	____/____/____
**Name:**		**Tester:**		

Instructions: Safety first, no task should be performed if either the participant or tester deem it unsafe. All participants should be tested in a well fitting prosthesis. Do not motivate or give performance tips to the participant. Trial 3 is used only in the case of a disqualification or fall during Trials 1&2. Record the Best Test Time/Points result between the 2 trials for each test in the conversion column. Use the table below to convert the Best Test Time/Point result to the Test Score. The sum of the four Test Scores will provide the Total CHAMP Score.

**Lower Limb Status**	**Intact**	**Trans-tibial**	**Knee disartic**	**Trans-femoral**	**Other (specify)** ____________
**Left Limb**	1	2	3	4	5
**Right Limb**	1	2	3	4	5

**Item**		**Trial 1**	**Trial 2**	**Trial 3**	**Conversion**	
		**Test Time/Point**	**Test Time/Point**	**Test Time/Point**	**Best Test Time/Point**	**Test Score**
**1. Single Limb Stance (seconds)** Arms crossed, foot raised a min. 15.2 cm	L				(Combined time for Left & Right SLS)	
off the floor maintaining single limb stance **30 sec. max each limb**	R					
**2. Edgren Side Step Test (points)** Record the number of points for each one meter interval side-stepped in 10 second. **If trial disqualified record 0**						
**3. T-Test (seconds)** Record the time in seconds to complete the course. **If trial disqualified record 0**						
**4. Illinois Agility Test (seconds)** Record the time in seconds to complete the course. **If trial disqualified record 0**						
					**Total CHAMP Score**	____/40

Source: Gaunaurd, Ignacio A., "The Comprehensive High-level Activity Mobility Predictor (CHAMP): A Performance-based Assessment Instrument to Quantify High-level Mobility in Service Members with Traumatic Lower Limb Loss" (2012). Open Access Dissertations. Paper 712.

Client ID

28755

OPUS: Health Quality of Life Index

**Note: For the questions below, the term "physical condition" refers to the reason you use an orthotic or prosthetic device.**	Not at all	A little	A fair amount	A great deal	Excessively
1. How much do you keep to yourself to avoid people's reactions to a missing body part or your need for a device?	○	○	○	○	○
2. To what extent do you find that people's attitudes toward your physical condition are insulting?	○	○	○	○	○
3. To what extent are you prevented from doing what you want to do because of social attitudes, the law, or environmental barriers?	○	○	○	○	○
4. How much does pain interfere with your activities (including both work outside the home and household duties)?	○	○	○	○	○
5. To what extent do you accomplish less than you would like because of your physical condition?	○	○	○	○	○
6. To what extent do you accomplish less than you would like because of emotional problems?	○	○	○	○	○
7. How much does your physical condition restrict your ability to run errands?	○	○	○	○	○
8. How much does your physical condition restrict your ability to pursue a hobby?	○	○	○	○	○
9. How much does your physical condition restrict your ability to do chores?	○	○	○	○	○
10. How much does your physical condition restrict your ability to do paid work?	○	○	○	○	○
11. To what extent have you cut down on work or other activities because of your physical condition?	○	○	○	○	○
12. To what extent have you cut down on work or other activities because of emotional problems?	○	○	○	○	○

© Rehabilitation Institute of Chicago, 2014

28755

Client ID

OPUS: Health Quality of Life Index

**During the past week, how often have you...**	All the time	Most of the time	Some of the time	A little of the time	None of the time
13. felt full of life?	O	O	O	O	O
14. felt calm and peaceful?	O	O	O	O	O
15. had a lot of energy?	O	O	O	O	O
16. been happy?	O	O	O	O	O
17. been very nervous?	O	O	O	O	O
18. felt so down in the dumps that nothing could cheer you up?	O	O	O	O	O
19. felt downhearted and depressed?	O	O	O	O	O
20. felt worn out?	O	O	O	O	O
21. felt tired?	O	O	O	O	O
22. been easily bothered or upset?	O	O	O	O	O
23. had difficulty concentrating or paying attention?	O	O	O	O	O

63125

Subject ID: _______________

Date: _______________

OPUS: Lower-Extremity Functional Status Measure

How easy, or difficult, is it for you to:	Very easy	Easy	Slightly difficult	Very difficult	Cannot do this activity	Do you typically wear an orthotic or prosthetic device to perform this activity?	
						No	Yes
1. Get into and out of the tub or shower	O	O	O	O	O	O	O
2. Dress your lower body	O	O	O	O	O	O	O
3. Get on and off the toilet	O	O	O	O	O	O	O
4. Get up from the floor	O	O	O	O	O	O	O
5. Balance while standing	O	O	O	O	O	O	O
6. Stand for one-half hour	O	O	O	O	O	O	O
7. Pick up an object from floor while standing	O	O	O	O	O	O	O
8. Get up from a chair	O	O	O	O	O	O	O
9. Get into and out of a car	O	O	O	O	O	O	O
10. Walk around indoors	O	O	O	O	O	O	O
11. Walk outside on uneven ground	O	O	O	O	O	O	O
12. Walk in bad weather (e.g., rain, snow, wind)	O	O	O	O	O	O	O
13. Walk up to two hours	O	O	O	O	O	O	O
14. Walk up a steep ramp	O	O	O	O	O	O	O
15. Get on and off an escalator	O	O	O	O	O	O	O
16. Climb one flight of stairs with a rail	O	O	O	O	O	O	O
17. Climb one flight of stairs without a rail	O	O	O	O	O	O	O
18. Run one block	O	O	O	O	O	O	O
19. Carry a plate of food while walking	O	O	O	O	O	O	O
20. Put on and take off orthosis or prosthesis	O	O	O	O	O		

© Rehabilitation Institute of Chicago, 2014

20694

Subject ID: _______________

Date: _______________

OPUS: Satisfaction With Device and Services

Please mark the response that most closely reflects your opinion.

	Strongly Agree	Agree	Neither Agree nor Disagree	Disagree	Strongly Disagree	Don't Know / Not Applicable
1. My prosthesis / orthosis fits well	○	○	○	○	○	○
2. The weight of my prosthesis / orthosis is manageable	○	○	○	○	○	○
3. My prosthesis / orthosis is comfortable throughout the day	○	○	○	○	○	○
4. It is easy to put on my prosthesis / orthosis	○	○	○	○	○	○
5. My prosthesis / orthosis looks good	○	○	○	○	○	○
6. My prosthesis / orthosis is durable	○	○	○	○	○	○
7. My clothes are free of wear and tear from my prosthesis / orthosis	○	○	○	○	○	○
8. My skin is free of abrasions and irritations	○	○	○	○	○	○
9. My prosthesis / orthosis is pain free to wear	○	○	○	○	○	○
10. I can afford the out-of-pocket expenses to purchase and maintain my prosthesis / orthosis	○	○	○	○	○	○
11. I can afford to repair or replace my prosthesis / orthosis as soon as needed	○	○	○	○	○	○
12. I received an appointment with a prosthetist / orthotist within a reasonable amount of time	○	○	○	○	○	○
13. I was shown the proper level of courtesy and respect by the staff	○	○	○	○	○	○
14. I waited a reasonable amount of time to be seen	○	○	○	○	○	○
15. Clinic staff fully informed me about equipment choices	○	○	○	○	○	○
16. The prosthetist / orthotist gave me the opportunity to express my concerns regarding my equipment	○	○	○	○	○	○
17. The prosthetist / orthotist was responsive to my concerns and questions	○	○	○	○	○	○
18. I am satisfied with the training I received in the use and maintenance of my prosthesis / orthosis	○	○	○	○	○	○
19. The prosthetist / orthotist discussed problems I might encounter with my equipment	○	○	○	○	○	○
20. The staff coordinated their services with my therapists and doctors	○	○	○	○	○	○
21. I was a partner in decision-making with clinic staff regarding my care and equipment	○	○	○	○	○	○

© Rehabilitation Institute of Chicago, 2014

Scoring Guide for the Orthotics and Prosthetics Users Survey

OPUS Lower Extremity Functional Status Measure (20 items)

The response to each item should be scored as follows:

4=Very Easy
3= Easy
2=Slightly difficult
1=Very difficult
0=Cannot do this activity

OPUS LE Total Score is the sum of the scores for the 20 items (0 – 80).
A higher score indicates a better outcome.

Use this table to convert raw score to Rasch Measure (0 – 100 scale).

To estimate an equal-interval measure from the sum of all items for patients with no missing data, find the value in the SCORE column and read across to find its MEASURE. MEASURE is scored to range from 0 (the lowest possible score) to 100 (the highest possible score).

TABLE OF MEASURES ON TEST OF 20 ITEM OPUS LE FUNCTIONAL STATUS MEASURE

SCORE	MEASURE	S.E.	SCORE	MEASURE	S.E.	SCORE	MEASURE	S.E.
0	.00E	13.13	27	39.89	2.11	54	56.28	2.19
1	8.67	7.21	28	40.51	2.10	55	56.95	2.21
2	13.72	5.16	29	41.12	2.09	56	57.64	2.23
3	16.76	4.28	30	41.73	2.08	57	58.33	2.25
4	18.99	3.77	31	42.33	2.07	58	59.04	2.27
5	20.79	3.44	32	42.92	2.07	59	59.77	2.30
6	22.31	3.21	33	43.51	2.06	60	60.51	2.32
7	23.66	3.03	34	44.10	2.06	61	61.27	2.35
8	24.88	2.90	35	44.69	2.06	62	62.04	2.38
9	26.00	2.79	36	45.28	2.05	63	62.85	2.42
10	27.05	2.70	37	45.86	2.05	64	63.67	2.46
11	28.03	2.62	38	46.45	2.05	65	64.53	2.50
12	28.96	2.56	39	47.04	2.06	66	65.42	2.56
13	29.85	2.51	40	47.63	2.06	67	66.34	2.62
14	30.71	2.46	41	48.21	2.06	68	67.32	2.68
15	31.53	2.41	42	48.81	2.07	69	68.35	2.76
16	32.33	2.38	43	49.40	2.07	70	69.45	2.86
17	33.10	2.34	44	50.00	2.08	71	70.63	2.97
18	33.85	2.31	45	50.60	2.08	72	71.90	3.10
19	34.58	2.28	46	51.21	2.09	73	73.31	3.27
20	35.29	2.25	47	51.82	2.10	74	74.89	3.47
21	35.99	2.23	48	52.43	2.11	75	76.69	3.74
22	36.67	2.20	49	53.05	2.12	76	78.82	4.11
23	37.34	2.18	50	53.68	2.13	77	81.46	4.65
24	37.99	2.16	51	54.32	2.15	78	85.02	5.55
25	38.64	2.14	52	54.96	2.16	79	90.75	7.58
26	39.27	2.13	53	55.62	2.18	80	100.00E	13.38

Using the Key Forms

For each Key Form, items are arrayed from hardest to endorse at the top to easiest to endorse at the bottom. Persons are arrayed from those with the lowest level of functioning (or quality of life or satisfaction) at the left to those with the highest level of functioning (or quality of life or satisfaction) at the right. This Key Form allows clinicians to estimate an individual's measure with missing responses to items. Clinicians can use the Key Form to estimate patients' measures and to look for unexpected responses.

KEY FORM FOR THE 20 ITEM OPUS LOWER EXTREMITY FUNCTIONAL STATUS MEASURE

```
EXPECTED SCORE: MEAN  (Rasch-score-point threshold, ":" indicates Rasch-half-point threshold) (ILLUSTRATED
BY AN OBSERVED CATEGORY)
0    10    20    30    40    50    60    70    80    90   100
|-----+-----+-----+-----+-----+-----+-----+-----+-----+-----|  NUM   ITEM
0                           0   :  1  :  2   :   3   :   4  4   30 18.run 1 block
|                                                           |
|                                                           |
0                     0  :   1  :  2  :   3    :   4        4   25 13.walk 2 hours
|                                                           |
0                    0  :  1  :  2   :   3    :  4          4   29 17.climb stairs w/o rail
|                                                           |
0                  0  :   1  :  2  :   3    :   4           4   26 14.walk up steep ramp
0                  0  :  1  :  2   :   3    :  4            4   24 12.walk in bad weather
0                 0  :  1  :  2   :   3    :  4             4   23 11.walk on uneven ground
|                                                           |
0               0  :   1  :  2  :   3    :   4              4   16 4.get up from floor
|                                                           |
0              0  :   1  :  2  :   3    :   4               4   31 19.walk with food
0              0  :  1  :  2   :   3    :   4               4   27 15.get on/off escalator
0              0  :  1  :  2   :   3    :  4                4   18 6.stand ½ hour
0             0  :   1  :  2   :  3    :   4                4   19 7.pick up object while standing
0             0  :   1  :  2  :   3    :   4                4   28 16.climb stairs w/rail
|                                                           |
0            0  :   1 :   2  :   3    :   4                 4   13 1.get in/out of tub or shower
|                                                           |
0          0  :   1  :  2  :   3    :   4                   4   17 5.balance while standing
0         0  :   1  :  2  :   3    :   4                    4   21 9.get in/out of car
0         0  :  1  :  2   :   3    :   4                    4   20 8.get up from chair
|                                                           |
0       0  :   1  :  2  :   3    :   4                      4   14 2.dress lower body
0       0  :   1 :   2  :   3    :   4                      4   22 10.walk indoors
0       0  :  1  :  2   :   3    :   4                      4   32 20.put on/take off pro./orth.
0      0  :   1  :  2  :   3    :   4                       4   15 3.get on/off toilet
|-----+-----+-----+-----+-----+-----+-----+-----+-----+-----|  NUM   ITEM
0    10    20    30    40    50    60    70    80    90   100

                1111 1121323322221 1
3    1    2 136342239826755719094230658551252 1  1 3        3  PERSON
            T      S       M       S      T
0                10 20 30 50 70 80 90            99            PERCENTILE
```

The distance between scale points is equal-interval. The scale at the top and bottom of the key ranges from 0 (lowest possible score) to 100 (highest possible score). To use the Key Form, circle a patient's responses to each item. We do not expect responses to deviate more than a single response level from an adjacent item. Draw a vertical line at a point midway between the majority of the responses; the point where this line intersects the horizontal axis is the estimated measure for that person.

OPUS Health Quality of Life Index (23 items)

The response to items 1-12 should be scored as follows:

4=Not at all
3= A little
2=A fair amount
1=A great deal
0=Excessively

The response to items 13-16 should be scored as follows:

4=All of the time
3=Most of the time
2=Some of the time
1=A little of the time
0=None of the time

The response to items 17-23 should be scored as follows:

0=All of the time
1=Most of the time
2=Some of the time
3=A little of the time
4=None of the time

The OPUS Health Quality of Life Score is the sum of the scores for the 23 items (0 – 92).
A higher score indicates a better outcome.
Use this table to convert raw score to Rasch Measure (0 – 100 scale).

TABLE OF MEASURES ON TEST OF 23 ITEM OPUS HEALTH QUALITY OF LIFE

SCORE	MEASURE	S.E.	SCORE	MEASURE	S.E.	SCORE	MEASURE	S.E.
0	.00E	15.48	31	42.28	1.92	62	55.20	1.97
1	10.22	8.49	32	42.71	1.91	63	55.66	1.99
2	16.11	6.01	33	43.14	1.90	64	56.13	2.01
3	19.58	4.92	34	43.57	1.89	65	56.61	2.03
4	22.05	4.28	35	43.99	1.88	66	57.10	2.05
5	23.98	3.84	36	44.40	1.88	67	57.60	2.07
6	25.57	3.52	37	44.82	1.87	68	58.12	2.10
7	26.93	3.28	38	45.23	1.87	69	58.64	2.13
8	28.12	3.09	39	45.64	1.86	70	59.19	2.16
9	29.19	2.93	40	46.05	1.86	71	59.75	2.20
10	30.15	2.80	41	46.45	1.85	72	60.33	2.24
11	31.04	2.68	42	46.86	1.85	73	60.93	2.28
12	31.86	2.59	43	47.26	1.85	74	61.55	2.33
13	32.62	2.51	44	47.66	1.85	75	62.21	2.38
14	33.34	2.44	45	48.07	1.85	76	62.89	2.44
15	34.02	2.37	46	48.47	1.85	77	63.61	2.50
16	34.67	2.32	47	48.87	1.85	78	64.37	2.58
17	35.29	2.27	48	49.28	1.85	79	65.18	2.66
18	35.89	2.22	49	49.68	1.85	80	66.04	2.75
19	36.46	2.18	50	50.09	1.86	81	66.96	2.85
20	37.01	2.15	51	50.50	1.86	82	67.96	2.97
21	37.55	2.12	52	50.91	1.87	83	69.05	3.11
22	38.07	2.09	53	51.32	1.87	84	70.26	3.28
23	38.57	2.06	54	51.73	1.88	85	71.60	3.48
24	39.07	2.04	55	52.15	1.89	86	73.13	3.73
25	39.55	2.02	56	52.57	1.89	87	74.90	4.05
26	40.03	2.00	57	53.00	1.90	88	77.04	4.48
27	40.49	1.98	58	53.43	1.91	89	79.73	5.11
28	40.95	1.96	59	53.86	1.93	90	83.43	6.19
29	41.40	1.95	60	54.30	1.94	91	89.59	8.62
30	41.84	1.93	61	54.75	1.95	92	100.00E	15.56

Source: sralab.org/rehabilitation-measures

KEY FORM FOR THE 23 ITEM OPUS HEALTH QUALITY OF LIFE MEASURE

EXPECTED SCORE: MEAN (Rasch-score-point threshold, ":" indicates Rasch-half-point threshold) (ILLUSTRATED BY AN OBSERVED CATEGORY)

```
10    20     30     40     50     60     70     80     90
|------+------+------+------+------+------+------+------|  NUM   ITEM
0                  0   :    1  : 2  :  3    :   4       4   11  11.cut down on work-physical condition
0                  0   :    1  : 2  :  3    :   4       4   10  10.physical ability restricts-paid work
|                                                       |
|                                                       |
0                0   :    1  : 2  :  3    :   4         4    5  5.acomplish less-physical condition
0            0    :    1   :  2   :    3      :    4    4   15  15.have a lot of energy
0                0   :    1 :  2 :   3   :    4         4    7  7.physical ability restricts-errands
|                                                       |
0             0    :   1   :  2  :   3    :    4        4   21  21.feel tired
|                                                       |
0              0   :    1  : 2  :  3    :   4           4    8  8.physical ability restricts-hobby
0              0   :    1  : 2  :  3    :   4           4    9  9.physical ability restricts-chores
0            0   :    1  :   2  :   3    :    4         4   20  20.feel worn out
0          0   :    1   :  2   :    3      :     4      4   13  13.feel full of life
0             0   :   1  :  2 :  3    :    4            4    4  4.pain interfere w.activities
|                                                       |
0        0    :    1   :  2   :    3      :    4        4   14  14.feel calm
0        0   :     1  :   2   :    3     :     4        4   16  16.been happy
|                                                       |
0         0   :    1  :  2   :   3    :   4             4   22  22.easily upset
0         0   :    1  :  2   :   3    :   4             4   17  17.been very nervous
0          0   :    1  : 2  :  3    :   4               4    3  3.prevented from doing what you like
0        0    :   1   :  2  :   3    :    4             4   23  23.difficulty paying attention
|                                                       |
|                                                       |
0        0    :   1  :  2 :  3    :    4                4   12  12.cut down on work-emotional condition
0       0   :    1  :  2   :   3    :   4               4   19  19.feel downhearted
0        0   :    1  : 2  :  3    :   4                 4    6  6.acomplish less-emotional condition
|                                                       |
|                                                       |
0     0   :    1   :  2  :   3    :    4                4   18  18.down in the dumps
|                                                       |
|                                                       |
0     0   :    1  : 2  :  3    :   4                    4    2  2.insulted by others attitudes
0     0   :    1  : 2  :  3    :   4                    4    1  1.keep to yourself to avoid reactions
|------+------+------+------+------+------+------+------|  NUM   ITEM
10    20     30     40     50     60     70     80     90

                       11122213334332111 1
           1     21218716053638816111815751614 2   31   4  PERSON
                   T      S     M     S      T
           0           10 20 30 60 80 90           99      PERCENTILE
```

Satisfaction With Device and Services (21 item survey)

The response to each item should be scored as follows:

5=Strongly Agree
4= Agree
3=Neither agree nor disagree
2=Disagree
1=Strongly disagree
[don't know=6, not applicable=8 are missing value codes and are not included in the scoring]

Satisfaction With Device Score is the sum of the scores for items 1-11 (11 – 55).

Satisfaction With Services Score is the sum of the scores for items 12-21 (10 – 50).

A higher score indicates a better outcome for both measures.

Use these tables to convert raw score to Rasch Measure (0 – 100 scale).

TABLE OF MEASURES ON TEST OF 11 ITEM SATISFACTION WITH DEVICE

SCORE	MEASURE	S.E.	SCORE	MEASURE	S.E.	SCORE	MEASURE	S.E.
11	.00E	16.92	26	37.54	2.75	41	50.75	3.32
12	11.30	9.33	27	38.35	2.72	42	52.00	3.48
13	17.87	6.60	28	39.14	2.70	43	53.38	3.66
14	21.69	5.37	29	39.93	2.70	44	54.92	3.87
15	24.38	4.63	30	40.72	2.70	45	56.65	4.11
16	26.45	4.14	31	41.51	2.70	46	58.59	4.35
17	28.14	3.78	32	42.31	2.72	47	60.77	4.60
18	29.59	3.53	33	43.12	2.74	48	63.18	4.83
19	30.86	3.33	34	43.94	2.77	49	65.84	5.06
20	32.01	3.18	35	44.79	2.81	50	68.76	5.33
21	33.06	3.06	36	45.66	2.86	51	72.04	5.68
22	34.04	2.96	37	46.57	2.92	52	75.87	6.24
23	34.97	2.89	38	47.52	3.00	53	80.73	7.25
24	35.85	2.83	39	48.52	3.09	54	88.19	9.72
25	36.71	2.78	40	49.59	3.20	55	100.00E	17.09

TABLE OF MEASURES ON TEST OF 10 ITEM SATISFACTION WITH SERVICES

SCORE	MEASURE	S.E.	SCORE	MEASURE	S.E.	SCORE	MEASURE	S.E.
10	.00E	16.19	24	35.38	2.67	38	48.44	3.83
11	10.80	8.88	25	36.18	2.63	39	50.29	4.26
12	16.95	6.22	26	36.95	2.60	40	52.60	4.76
13	20.51	5.08	27	37.72	2.59	41	55.45	5.23
14	23.06	4.44	28	38.48	2.59	42	58.75	5.49
15	25.08	4.03	29	39.24	2.60	43	62.22	5.53
16	26.78	3.72	30	40.02	2.63	44	65.67	5.50
17	28.26	3.49	31	40.82	2.67	45	69.12	5.53
18	29.56	3.29	32	41.65	2.74	46	72.69	5.71
19	30.73	3.13	33	42.53	2.82	47	76.64	6.12
20	31.80	2.99	34	43.46	2.93	48	81.46	7.01
21	32.78	2.88	35	44.48	3.07	49	88.68	9.32
22	33.69	2.79	36	45.62	3.26	50	100.00E	16.34
23	34.55	2.72	37	46.91	3.51			

KEY FORM FOR THE 11 ITEM SATISFACTION WITH DEVICE MEASURE

```
EXPECTED SCORE: MEAN  (Rasch-score-point threshold, ":" indicates Rasch-half-point threshold) (ILLUSTRATED
BY AN OBSERVED CATEGORY)
10    20     30     40     50     60     70     80     90
|------+------+------+------+------+------+------+------|  NUM   ITEM
1             1    :   2  :  3  :   4        :      5   5   11  11.can afford to repair or replace
1             1   :    2  : 3  :    4        :      5   5   10  10.can afford out of pocket expenses
|                                                       |
|                                                       |
1          1    :   2  :  3  :   4        :      5      5    9  9.pain free to wear
|                                                       |
1         1   :    2 :  3  :    4        :     5        5    1  1.fits well
1         1   :    2 :  3  :    4        :     5        5    3  3.comfortable throughout the day
|                                                       |
1        1   :    2 :  3  :    4        :     5         5    8  8.skin is free of abrasions & irritation
1       1   :    2  : 3  :    4        :      5         5    7  7.clothes are free of wear & tear
|                                                       |
1     1    :   2  : 3  :    4        :      5           5    5  5.looks good
1     1   :    2 :  3  :    4        :     5            5    2  2.weight is manageable
|                                                       |
1   1    :   2  :  3  :   4        :      5             5    4  4.easy to put on
1  1    :   2  :  3  :    4        :     5              5    6  6.durable
|------+------+------+------+------+------+------+------|  NUM   ITEM
10    20     30     40     50     60     70     80     90

                           1 1  1
1            1   1 2133926398285219 1 1412 2     2  1  1   PERSON
              T      S       M      S      T
0                     10 40 60 70 80  90          99      PERCENTILE
```

KEY FORM FOR THE 10 ITEM SATISFACTION WITH SERVICES MEASURE

```
EXPECTED SCORE: MEAN  (Rasch-score-point threshold, ":" indicates Rasch-half-point threshold) (ILLUSTRATED
BY AN OBSERVED CATEGORY)
0    10    20    30    40    50    60    70    80    90
|-----+-----+-----+-----+-----+-----+-----+-----+-----|  NUM   ITEM
1              1   :  2  : 3 :    4         :     5   5    3  14.reasonable wait time
1              1  :   2 : 3  :    4         :    5    5   10  21.I was a partner in decision making
1             1  :   2 : 3  :    4         :    5     5    4  15.informed of equipment choices
1            1   :  2  : 3 :    4         :     5     5    1  12.appointment in reasonable time
1            1   :  2  : 3 :    4         :     5     5    8  19.discussed problems
1            1  :   2 : 3  :    4         :     5     5    7  18.satisfied with training received
1           1   :  2  : 3 :    4         :     5      5    9  20.coordinated services with therapists &
|                                                     |          doctors
1          1   :  2  : 3 :    4         :     5       5    5  16.opportunity to express concerns
1          1   :  2 : 3  :    4         :     5       5    6  17.responsive to concerns and questions
|                                                     |
|                                                     |
1    1   :  2  : 3 :    4         :     5             5    2  13.shown courtesy & respect
|-----+-----+-----+-----+-----+-----+-----+-----+-----|  NUM   ITEM
0    10    20    30    40    50    60    70    80    90

                               2                   11
                      1 2223427325 314 5 4114 5  5   78  PERSON
                  T         S           M           S
                      0    10 20 40 50     60   70  80 99PERCENTILE
```

<u>OPUS Upper Extremity Functional Status Measure (28 items)</u>

4=Very Easy
3= Easy
2=Slightly difficult
1=Very difficult
0=Cannot do this activity
[not applicable can be coded 8. This is a missing value code and is not included in the scoring]

OPUS UE Total score is the sum of the scores for the 28 items (0 – 112).

A higher score indicates a better outcome.
The table of measures and key form for the UE Functional Status Measure is being developed.

<u>Delivery & Follow Up Satisfaction Survey (8 items)</u>

<u>The response to each item should be scored as follows:</u>

5=Very Satisfied
4= Somewhat satisfied
3=Neither satisfied nor dissatisfied
2=Somewhat dissatisfied
1=Very Dissatisfied

A higher score indicates a better outcome.
This is a new survey and currently there is no table of Rasch measures or key form.

Date_______________ **Tester Initials:________** **Subject ID:_______**

The Modified Falls Efficacy Scale

On a scale of 0 to 10, please rate how confident you are that you can do each of these activities without falling, with 0 meaning "not confident/not sure at all", 5 being "fairly confident/fairly sure", and 10 being "completely confident/completely sure".

Note:

* If you have stopped doing the activity at least partly because of being afraid of falling, score a 0

* If you have stopped an activity purely because of a physical problem, leave that item blank (these items are not included in the calculation of the average MFES score).

* If you do not currently do the activity for other reasons, please rate that item based on how you perceive you would rate it if you had to do the activity today.

		Not Confident					Fairly Confident					Completely Confident
	**Activity**	**0**	**1**	**2**	**3**	**4**	**5**	**6**	**7**	**8**	**9**	**10**
1.	Get dressed and undressed											
2.	Prepare a simple meal											
3.	Take a bath or a shower											
4.	Get in/out of a chair											
5.	Get in/out of bed											
6.	Answer the door or telephone											
7.	Walk around the inside of your house											
8.	Reach into cabinets or closet											
9.	Light housekeeping											
10.	Simple shopping											
11.	Using public transport											
12.	Crossing roads											
13.	Light gardening or hanging out the washing *											
14.	Using front or rear steps at home											

* Rate most commonly performed of these activities

Score/Item Rated= _____/_____

Average= _____

Subject ID: ______________

Date: ______________

PATIENT HEALTH QUESTIONNAIRE-9 (PHQ-9)

**Over the last 2 weeks, how often have you been bothered by any of the following problems?** *(Use "✔" to indicate your answer)*	**Not at all**	**Several days**	**More than half the days**	**Nearly every day**
**1.** Little interest or pleasure in doing things	0	1	2	3
**2.** Feeling down, depressed, or hopeless	0	1	2	3
**3.** Trouble falling or staying asleep, or sleeping too much	0	1	2	3
**4.** Feeling tired or having little energy	0	1	2	3
**5.** Poor appetite or overeating	0	1	2	3
**6.** Feeling bad about yourself — or that you are a failure or have let yourself or your family down	0	1	2	3
**7.** Trouble concentrating on things, such as reading the newspaper or watching television	0	1	2	3
**8.** Moving or speaking so slowly that other people could have noticed? Or the opposite — being so fidgety or restless that you have been moving around a lot more than usual	0	1	2	3
**9.** Thoughts that you would be better off dead or of hurting yourself in some way	0	1	2	3

FOR OFFICE CODING ___0___ + ______ + ______ + ______

=Total Score: ______

If you checked off any problems, how difficult have these problems made it for you to do your work, take care of things at home, or get along with other people?

**Not difficult at all**	**Somewhat difficult**	**Very difficult**	**Extremely difficult**
☐	☐	☐	☐

Developed by Drs. Robert L. Spitzer, Janet B.W. Williams, Kurt Kroenke and colleagues, with an educational grant from Pfizer Inc. No permission required to reproduce, translate, display or distribute.

Community Participation Indicators

This survey is voluntary. If you choose to participate, your information will be kept private. Your name will never be linked to any of the information you share.

Shade circles like this: ●
Not like this: ⊠ ⊘

The statements below describe many of the ways that people participate in society. For each item, tell us:

1) How often you do the activity,
2) If the activity is important to you, and
3) If you feel you are doing the activity enough, too much, or not enough.

Subject ID:________________

Date:____________________

1. How often? --> 2. Important? --> 3. Doing enough?

In a typical week, how many days do you:	None	1-2 Days	3-4 Days	5-6 Days	7 Days	Is this activity important to you? No	Yes	Are you doing this activity: Enough	Not Enough	Too Much
Get out and about	○	○	○	○	○	○	○	○	○	○
Spend time with family	○	○	○	○	○	○	○	○	○	○
Keep in touch with family by phone or Internet	○	○	○	○	○	○	○	○	○	○
Spend time with friends	○	○	○	○	○	○	○	○	○	○
Keep in touch with friends by phone or Internet	○	○	○	○	○	○	○	○	○	○
Go to parties, out to dinner, or other social activities	○	○	○	○	○	○	○	○	○	○
Spend time with a significant other or intimate partner	○	○	○	○	○	○	○	○	○	○

7532

Shade circles like this: ●
Not like this: ⊠ ⊘

For each item, tell us:

1) How often you do the activity,
2) If the activity is important to you, and
3) If you feel you are doing the activity enough, too much, or not enough.

Subject ID:________________

Date:____________________

1. How often? --> 2. Important? --> 3. Doing enough?

In a typical week, how many hours do you:	None	1-4 Hours	5-9 Hours	10-19 Hours	20-34 Hours	35 or more Hours	Is this activity important to you? No	Yes	Are you doing this activity: Enough	Not Enough	Too Much
Work for money	○	○	○	○	○	○	○	○	○	○	○
Cook, clean, and look after your home	○	○	○	○	○	○	○	○	○	○	○
Manage household bills and expenses	○	○	○	○	○	○	○	○	○	○	○
Look after children or provide care for a loved one	○	○	○	○	○	○	○	○	○	○	○
Go to classes or participate in learning activities	○	○	○	○	○	○	○	○	○	○	○
Volunteer	○	○	○	○	○	○	○	○	○	○	○

7532

Community Participation Indicators

Shade circles like this: ●
Not like this: ⊠ ⊘

For each item, tell us:
1) How often you do the activity,
2) If the activity is important to you, and
3) If you feel you are you are doing the activity enough, too much, or not enough.

Subject ID:________________

Date:____________________

1. How often? --> 2. Important? --> 3. Doing enough?

In a typical month, how many times do you:	None	Once	2 Times	3 Times	4 Times	5 or More Times	Is this activity important to you? No	Yes	Are you doing this activity: Enough	Not Enough	Too Much
Participate in religious or spiritual activities	○	○	○	○	○	○	○	○	○	○	○
Go to support groups or self-help meetings	○	○	○	○	○	○	○	○	○	○	○
Engage in hobbies or leisure activities	○	○	○	○	○	○	○	○	○	○	○
Go to movies, sporting events or entertainment events	○	○	○	○	○	○	○	○	○	○	○
Exercise, participate in sports or active recreation	○	○	○	○	○	○	○	○	○	○	○
Participate in community clubs or organizations	○	○	○	○	○	○	○	○	○	○	○
Participate in civic or political activities	○	○	○	○	○	○	○	○	○	○	○

7532

Subject ID: ____________

Shade circles like this: ⬣
Not like this: ☒ ⌀

Date: ________________

Please mark the choice that most closely reflects your opinion:

	All the time	Frequently	Sometimes	Seldom	Almost never
1. I live my life the way that I want	○	○	○	○	○
2. People try to put limits on me	○	○	○	○	○
3. I participate in a variety of activities	○	○	○	○	○
4. I am uncomfortable participating in community activities	○	○	○	○	○
5. I spend time doing things that improve my community	○	○	○	○	○
6. I participate in activities that I choose	○	○	○	○	○
7. I spend time helping others	○	○	○	○	○
8. I count as a person in society	○	○	○	○	○
9. I have the freedom to make my own decisions	○	○	○	○	○
10. I live my life fully	○	○	○	○	○
11. I regularly seek out new challenges	○	○	○	○	○
12. I have reliable access to a telephone	○	○	○	○	○
13. I have a say on decisions in my community	○	○	○	○	○
14. I have choices about the activities I do	○	○	○	○	○
15. I actively pursue my dreams and desires	○	○	○	○	○
16. I do things that are important to me	○	○	○	○	○
17. People have high expectations of me	○	○	○	○	○
18. I am able to go out and have fun	○	○	○	○	○
19. I contribute to society	○	○	○	○	○
20. I have opportunities to make new friends	○	○	○	○	○
21. I speak up for myself	○	○	○	○	○
22. People speak to me disrespectfully	○	○	○	○	○
23. I take responsibility for my own life	○	○	○	○	○
24. I have good job opportunities	○	○	○	○	○
25. People underestimate me	○	○	○	○	○

Subject ID: _____________

Date: _________________

Shade circles like this: ●
Not like this: ⊠ ⊘

Please mark the choice that most closely reflects your opinion:

	All the time	Frequently	Sometimes	Seldom	Almost never
26. I assume leadership roles in organizations	○	○	○	○	○
27. I am welcome in my community	○	○	○	○	○
28. I am treated equally	○	○	○	○	○
29. I have reliable access to community services	○	○	○	○	○
30. I do important things with my life	○	○	○	○	○
31. My community respects me the way that I am	○	○	○	○	○
32. I have influence in my community	○	○	○	○	○
33. I am in control of my own life	○	○	○	○	○
34. I am ignored	○	○	○	○	○
35. I feel safe participating in community activities	○	○	○	○	○
36. I am treated as a valued member of society	○	○	○	○	○
37. People see my potential	○	○	○	○	○
38. I have access to reliable transportation	○	○	○	○	○
39. I have reliable access to the Internet	○	○	○	○	○
40. I have control over how I spend my time	○	○	○	○	○
41. People listen to what I say	○	○	○	○	○
42. I participate in activities when I want	○	○	○	○	○
43. I am uncomfortable participating in public meetings	○	○	○	○	○
44. I am treated like a human being	○	○	○	○	○
45. People count on me	○	○	○	○	○
46. I contribute to the well-being of my community	○	○	○	○	○
47. I am actively involved in my community	○	○	○	○	○
48. It is hard for me to get information about community services	○	○	○	○	○

Source: sralab.org/rehabilitation-measures

CPI Scoring Guide

Sample Norms and Frequencies

The following pairs of tables provide (1) sample norms and frequencies and (2) Key Forms for the two measures comprising the Community Participation Indicators: Involvement in Life Situations and Control over Participation. Inclusion criteria for the sample were a self-identified disability, age 18 years and older, and ability to read and speak English. We recruited respondents with self-identified disabilities from multiple sources in order to obtain diverse sample, including an online panel generation company, recent inpatients of the Rehabilitation Institute of Chicago (RIC), attendees of several disability community events, independent living center and disability community organizations, a registry of current and former patients from RIC, Veterans Administration outpatients, and traumatic brain injury and spinal cord injury Model Systems collaborators. The convenience sample totaled 1,163 respondents. The median age was 53 years (S.D. = 17 years). Women comprised 49% of the sample. Race distribution included Caucasian (72%), African-American (12%), and Hispanic (7%) participants. Items were completed by self-report for 72% of the sample and interviewer-assisted for others. Self-reported disability severity was characterized as slight by 10% of the sample, moderate by 37%, somewhat severe by 38% and very severe by 14%. Respondents could identify multiple impairment categories. Self-reported impairment or condition categories included physical (63%), emotional (23%), hearing (14%), vision (13%), learning (11%) and communicative disabilities (9%). Marital status included married (38%), never married (23%), widowed (14%), divorced (9%), unmarried couple (5%), and separated (2%). The most frequently reported sources of income were Social Security Disability Insurance (23%), other household members' employment (21%), employment (19%), and retirement income (13%). Most participants reported living in a private residence (93%). Assistive device use was reported by 51% of the sample.

Table were produced with Winsteps software.

To estimate an equal-interval measure from the sum of all items for patients with no missing data, find the value in the SCORE column and read across to find its MEASURE. MEASURE is scored to range from 0 (the lowest possible score) to 100 (the highest possible score). These norms should be interpreted with caution; they are provisional and reflect the sampling strategy.

Using the Key Forms

For each Key Form, items are arrayed from easiest to endorse at the top to hardest to endorse at the bottom. Persons are arrayed from those with the lowest level of involvement or control at the left to those with the highest level of involvement or control at the right. This Key Form allows clinicians to estimate an individual's measure with missing responses to items. Clinicians can use the Key Form to estimate patients' measures and to look for unexpected responses.

The distance between scale points is equal-interval. The scale at the top and bottom of the key ranges from 0 (lowest possible score) to 100 (highest possible score). To use the Key Form, circle a patient's responses to each item. We do not expect responses to deviate more than a single response level from an adjacent item. Draw a vertical line at a point midway between the majority of the responses; the point where this line intersects the horizontal axis is the estimated measure for that person.

Abbreviations are: Q=quartile; S=standard deviation; T=2 standard deviations; M=median; SE=standard error.

Copyright 2011 Rehabilitation Institute of Chicago

Source: sralab.org/rehabilitation-measures

```
TABLE 2.2 F:\RICconsult\CPI\InvolvementR InvolvementRptg2.out  Apr 25 11:45 2011
INPUT: 1085 PERSON  48 ITEM  REPORTED: 1082 PERSON  14 ITEM  5 CATS WINSTEPS 3.71.0.1
------------------------------------------------------------------------------------
```

Involvement in Life Situations

```
EXPECTED SCORE: MEAN  (Rasch-score-point threshold, ":" indicates Rasch-half-point threshold)
(ILLUSTRATED BY AN OBSERVED CATEGORY)
10     20     30     40     50     60     70     80     90
|------+------+------+------+------+------+------+------|NUM  ITEM
1                1   :    2   :  3   :   4     :    5   5  5 I spend time doing things that improve my community
|                                                       |
|                                                       |
1              1   :    2   :  3   :   4     :    5     5 13 I have a say on decisions in my community
1              1   :    2   :  3   :   4     :    5     5 47 I am actively involved in my community
1             1    :    2  :   3   :   4    :    5      5 26 I assume leadership roles in organizations
|                                                       |
1            1    :    2  :   3  :    4    :    5       5 32 I have influence in my community
|                                                       |
|                                                       |
1          1   :    2   :  3   :   4     :   5          5  3 I participate in a variety of activities
1         1   :    2   :  3   :   4     :    5          5 46 I contribute to the well-being of my community
1         1   :    2   :  3   :   4     :    5          5 11 I regularly seek out new challenges
1        1    :    2  :   3   :   4    :    5           5 19 I contribute to society
|                                                       |
1       1   :    2   :  3   :   4     :   5             5 37 People see my potential
1      1    :    2   :  3   :   4    :    5             5  7 I spend time helping others
|                                                       |
1     1    :    2  :   3  :    4    :    5              5 30 I do important things with my
|                                                       |
1    1    :    2  :   3  :    4    :    5               5 35 I feel safe participating in community
|                                                       |
1  1    :    2   :  3   :   4    :    5                 5 45 People count on me
|------+------+------+------+------+------+------+------|NUM  ITEM
10     20     30     40     50     60     70     80     90
             1 21142433376676357523212 1 1 1
821   6  1 39 375142267051642479628604639164 45 5  6    48  PERSON
          T      S        M        S       T
0                10 20 30 50 60 80  90                99   PERCENTILE
```

TABLE OF MEASURES ON TEST OF 14 ITEM

SCORE	MEASURE	S.E.	SCORE	MEASURE	S.E.	SCORE	MEASURE	S.E.
14	.00E	17.83	33	41.51	2.81	52	56.38	2.93
15	11.42	9.59	34	42.30	2.79	53	57.26	2.97
16	17.80	6.73	35	43.08	2.77	54	58.17	3.02
17	21.51	5.50	36	43.85	2.76	55	59.10	3.07
18	24.17	4.80	37	44.62	2.75	56	60.07	3.13
19	26.27	4.34	38	45.38	2.74	57	61.08	3.19
20	28.02	4.01	39	46.14	2.74	58	62.14	3.27
21	29.55	3.77	40	46.90	2.73	59	63.25	3.36
22	30.91	3.58	41	47.65	2.73	60	64.43	3.47
23	32.15	3.43	42	48.41	2.74	61	65.69	3.60
24	33.30	3.31	43	49.17	2.74	62	67.06	3.76
25	34.37	3.21	44	49.93	2.75	63	68.56	3.96
26	35.39	3.13	45	50.70	2.76	64	70.25	4.21
27	36.35	3.06	46	51.48	2.78	65	72.18	4.55
28	37.28	3.00	47	52.26	2.80	66	74.48	5.02
29	38.17	2.95	48	53.06	2.82	67	77.38	5.74
30	39.03	2.91	49	53.86	2.84	68	81.40	6.98
31	39.88	2.87	50	54.68	2.87	69	88.19	9.84
32	40.70	2.84	51	55.52	2.90	70	100.00E	18.02

```
CURRENT VALUES, UMEAN=48.8192 USCALE=9.8961
TO SET MEASURE RANGE AS 0-100, UMEAN=48.8192 USCALE=9.8961
TO SET MEASURE RANGE TO MATCH RAW SCORE RANGE, UMEAN=41.3387 USCALE=5.5418
Predicting Score from Measure: Score = Measure * .8561 + -13.8122
Predicting Measure from Score: Measure = Score * 1.0859 + 18.4326
```


Source: sralab.org/rehabilitation-measures

```
TABLE 2.2 F:\RICconsult\CPI\ControlRptgSpx2. ControlRptg2.out  Apr 25 11:44 2011
INPUT: 1085 PERSON  48 ITEM  REPORTED: 1085 PERSON  13 ITEM  5 CATS WINSTEPS 3.71.0.1
--------------------------------------------------------------------------------------
```

Control over Participation

```
EXPECTED SCORE: MEAN  (Rasch-score-point threshold, ":" indicates Rasch-half-point threshold)
(ILLUSTRATED BY AN OBSERVED CATEGORY)
10     20      30      40      50      60      70      80      90
|-------+-------+-------+-------+-------+-------+-------+-------|NUM  ITEM
1                1    :     2   :    3  :     4      :     5    5 18 I am able to go out and have fun
1                1    :     2   :   3   :     4      :     5    5 15 I actively pursue my dreams and desires
1                1    :     2   :   3   :     4      :     5    5 20 I have opportunities to make new friends
|                                                               |
|                                                               |
1              1    :    2    :  3    :     4    :     5        5 10 I live my life fully
1             1     :   2    :   3    :   4      :     5        5  1 I live my life the way that I want
|                                                               |
1          1     :     2  :    3    :   4      :     5          5 42 I participate in activities when I want
1          1     :    2   :    3    :   4      :     5          5  6 I participate in activities that I choose
|                                                               |
1         1    :     2   :   3   :     4     :      5           5 16 I do things that are important to me
1        1    :     2   :   3   :     4      :     5            5 33 I am in control of my own life
|                                                               |
1      1    :    2    :   3   :     4     :     5               5 14 I have choices about the activities I do
1      1    :    2    :  3    :     4    :     5                5 40 I have control over how I spend my time
|                                                               |
1    1    :     2   :   3    :   4      :     5                 5  9 I have the freedom to make my own decisions
|                                                               |
|                                                               |
1 1    :     2   :   3   :     4      :     5                   5 23 I take responsibility for my own life
|-------+-------+-------+-------+-------+-------+-------+-------|NUM  ITEM
10     20      30      40      50      60      70      80      90
                  1 1112 453554536445733 222 3 2  3    24
2         22 15441058454700727190727227427961117227  1152PERSON
              T          S         M         S         T
0                      10 20 30 50 60 70 80     90     99PERCENTILE
```

TABLE OF MEASURES ON TEST OF 13 ITEM

SCORE	MEASURE	S.E.	SCORE	MEASURE	S.E.	SCORE	MEASURE	S.E.
13	.00E	17.50	31	40.79	2.92	49	56.73	3.15
14	11.15	9.40	32	41.66	2.90	50	57.77	3.20
15	17.39	6.61	33	42.52	2.88	51	58.84	3.25
16	21.04	5.43	34	43.37	2.87	52	59.95	3.32
17	23.68	4.76	35	44.22	2.87	53	61.10	3.39
18	25.78	4.32	36	45.06	2.86	54	62.31	3.48
19	27.55	4.01	37	45.90	2.86	55	63.59	3.58
20	29.11	3.78	38	46.74	2.87	56	64.96	3.71
21	30.51	3.60	39	47.59	2.88	57	66.42	3.86
22	31.78	3.46	40	48.44	2.89	58	68.03	4.05
23	32.97	3.35	41	49.31	2.90	59	69.81	4.29
24	34.09	3.25	42	50.18	2.92	60	71.84	4.62
25	35.15	3.18	43	51.06	2.94	61	74.24	5.08
26	36.17	3.11	44	51.96	2.97	62	77.25	5.78
27	37.14	3.06	45	52.87	3.00	63	81.36	6.99
28	38.09	3.01	46	53.80	3.03	64	88.23	9.78
29	39.01	2.98	47	54.75	3.06	65	100.00E	17.79
30	39.91	2.94	48	55.73	3.10			

```
CURRENT VALUES, UMEAN=48.2492 USCALE=9.7387
TO SET MEASURE RANGE AS 0-100, UMEAN=48.2492 USCALE=9.7387
TO SET MEASURE RANGE TO MATCH RAW SCORE RANGE, UMEAN=38.0896 USCALE=5.0641
Predicting Score from Measure: Score = Measure * .7751 + -11.4212
Predicting Measure from Score: Measure = Score * 1.2066 + 16.9082
```


Source: sralab.org/rehabilitation-measures
